# Microorganisms as Shapers of Human Civilization, from Pandemics to Even Our Genomes: Villains or Friends? A Historical Approach

**DOI:** 10.3390/microorganisms9122518

**Published:** 2021-12-06

**Authors:** Francisco Rodríguez-Frías, Josep Quer, David Tabernero, Maria Francesca Cortese, Selene Garcia-Garcia, Ariadna Rando-Segura, Tomas Pumarola

**Affiliations:** 1 Clinical Biochemistry Research Group, Department of Biochemistry, Vall d’Hebron Institut Recerca-Hospital Universitari Vall d’Hebron, Universitat Autònoma de Barcelona, 08035 Barcelona, Spain; maria.cortese@vhir.org (M.F.C.); selene.garcia@vhir.org (S.G.-G.); 2 Centro de Investigación Biomédica en Red de Enfermedades Hepáticas y Digestivas, Instituto de Salud Carlos III, 28029 Madrid, Spain; josep.quer@vhir.org; 3 Liver Pathology Unit, Departments of Biochemistry and Microbiology, Hospital Universitari Vall d’Hebron, Universitat Autònoma de Barcelona, 08035 Barcelona, Spain; a.rando@vhebron.net; 4 Liver Unit, Liver Disease Laboratory-Viral Hepatitis, Vall d’Hebron Institut Recerca, Hospital Universitari Vall d’Hebron, Vall d’Hebron Barcelona Hospital Campus, 08035 Barcelona, Spain; 5 Department of Microbiology, Hospital Universitari Vall d’Hebron, Vall d’Hebron Barcelona Hospital Campus, Passeig Vall d’Hebron 119-129, 08035 Barcelona, Spain; tpumarola@vhebron.net

**Keywords:** pandemics, plague, *Yersinia pestis*, influenza, COVID-19, SARS-CoV-2, microbiota, endogenous retrovirus, biotechnology, population genetics

## Abstract

Universal history is characterized by continuous evolution, in which civilizations are born and die. This evolution is associated with multiple factors, among which the role of microorganisms is often overlooked. Viruses and bacteria have written or decisively contributed to terrible episodes of history, such as the Black Death in 14th century Europe, the annihilation of pre-Columbian American civilizations, and pandemics such as the 1918 Spanish flu or the current COVID-19 pandemic caused by the coronavirus SARS-CoV-2. Nevertheless, it is clear that we could not live in a world without these tiny beings. Endogenous retroviruses have been key to our evolution and for the regulation of gene expression, and the gut microbiota helps us digest compounds that we could not otherwise process. In addition, we have used microorganisms to preserve or prepare food for millennia and more recently to obtain drugs such as antibiotics or to develop recombinant DNA technologies. Due to the enormous importance of microorganisms for our survival, they have significantly influenced the population genetics of different human groups. This paper will review the role of microorganisms as “villains” who have been responsible for tremendous mortality throughout history but also as “friends” who help us survive and evolve.

## 1. Introduction

*Everything about microscopic life is terribly upsetting. How can things so small be so important?*

Isaac Asimov

Since the Sumerian civilization developed writing [1], we have records that cover a period of 5000 years of human history, throughout which human society has been constantly evolving in the same way as living organisms. This evolution had included great changes that have sometimes taken place over a very short period of time and that have conditioned our history as a species. Many factors have been associated with these dramatic changes, including major inventions and discoveries such as agriculture, livestock, navigation, metals, writing itself; more recent discoveries, sch as the printing press, the steam engine; and modern discoveries, such as the Internet, social media, mass media, artificial intelligence, machine learning, etc., that were speculations from the world of science fiction only a few years ago. For instance, a world in which all computers could answer any question when they were interconnected was suggested in the short story “Answer” by Frederic Brown in 1954 [2]. Many of these changes were driven by natural human curiosity and the love of knowledge, philosophy, and science, and we might enter these on the positive side of history’s ledger, but history also contains many sad chapters, many of which have been caused by religious, economic, and/or political confrontations. Nevertheless, these instances are still marked as chapters in human history. Either way, all of these factors have shaped our history. However, other elements that are not as obvious but that have had an equal or even greater impact on the course of our history and even on the innermost part of us, our gene pool, exist. These additional causes are not usually included in history texts, and they are mediated by tiny, almost invisible beings: microorganisms. These tiny beings have caused millions of deaths, largely conditioning the most significant changes in our history, but they have also built us and probably all other life forms on this planet as well: villains and friends, that is the subject of this text. Similar to terrible villains, these beings have caused devastating pandemics that have been responsible for major demographic changes due to deaths in relatively short periods of time, some of which have been carried by other small but not so invisible beings, the vectors, such as mosquitoes [3] or fleas [4];however, these diseases have also been transmitted through the air or simply as fomites, in which the infectious agent is deposited on various surfaces and wait for an unsuspecting and unprotected host to unwittingly pick them up [5].

Large pandemics have been described since the beginning of our documented history. We have accurate data representing more than 2000 years. Thucydides in the 5th century BC in Athens [6], Galen in the 2nd century AD in Rome [7], Procopius in the 6th century AD in Byzantium [8], and Father Bartolomé de las Casas in the 16th century in the shameful chronicles of the near genocide in the Spanish possessions in the New World [9] are all examples of people who have recorded the pandemics that have occurred throughout our existence. The aim of this text is not to provide an exhaustive description of all these epidemics but to instead illustrate how these events caused by invisible enemies have shaped our history and have almost directed the evolution of our civilization. In less remote times, the great plagues of Europe are irreversibly linked to our history, as clearly exemplified by the Black Death in the 14th century [10]. Among the suffering that microorganisms have caused to humanity throughout history, this text discusses the following:

- 2.1. Pandemics Have Weakened empires. The First Records of Major Pandemics.
- 2.2. The “Black Death”, the Great Plague Pandemic of the 14th century. The World Changes at the Huge Cost of Human Lives.
- 2.2.1. Did the Plague Only Travel to Western Europe?
- 2.2.2. How did the Black Death Change History?
- 2.2.3. The Plague, a Scourge Much Older Than We Thought? A Terrible Traveling Companion for Millennia.
- 2.3. America “Ceases to Be an Island for Plagues” and Almost Succumbs to the Arrival of the Europeans. Acculturation or Assimilation? Facilitated by Microorganisms.
- 2.4. The 1918 Flu Pandemic: “the Spanish flu”.
- 2.4.1. Consequences of the 1918 Flu Pandemic.
- 2.5. Yet Another Tremendous Pandemic Disease: COVID-19, Coronavirus SARS-CoV-2, a “New Kid on the Block”.
- 2.5.1. New Opportunities Are Born from All Crises.
- 2.5.2. What about the Virus?
- 2.5.3. What Is the Origin of SARS-CoV-2?
- 2.5.4. What Is Being Changed Directly by the Effect of SARS –CoV-2?

Current scientific knowledge, including the sophisticated methods of molecular biology [4,11,12,13,14], allows us to rigorously explore those ancient events and go beyond the bibliographic data that are available, sequences of appearance, affected populations, and many other details. It seems clear how implacable these invisible enemies are, as the present SARS-CoV-2 pandemic has reminded us, having caused millions of deaths, with numbers increasing daily [15]. Nevertheless, as we will discuss later, the technological advances of recent decades have mitigated the consequences of the current pandemic caused by the SARS-CoV-2 virus, and this pandemic has fueled new methodologies to confront new pandemics. On the one hand, the rapid expansion of this pandemic can be attributed to the globalization of our society, with the practical disappearance of geographical borders due to the ease of fast travel. On the other hand, the same globalization has allowed scientists to collaborate in a type of planetary macro-neural network that is able to generate answers and solutions in record time. From a simplistic standpoint, it can be said that the greatest advantage that humans have over viruses is that a Korean virus, for example, cannot explain to a virus from Barcelona how to infect a patient, but a Korean scientist can collaborate with a colleague from Barcelona, and together, they can find an antiviral treatment or vaccine (adapted from *Sapiens: a Brief History of Humankind*, by Yuval Noah Harari, historian and philosopher) [16]. This globally shared knowledge has converged with obvious economic interest, making it possible to achieve new goals in record time. This pandemic has driven a vaccine revolution, with the development of highly effective vaccines being achieved in just one year [17] and the development of new diagnostic and genetic tools [18,19]. New invisible enemies will likely appear in the not-too-distant future, and in the meantime, this mindless but tenacious enemy has caused another great leap in our civilization, reshaping it as other microorganisms have before. The history of mankind has been widely and deeply affected by extremely lethal diseases that, when new to an area, initially have devastated the population. However, within a few years, these diseases can turn into childhood diseases to which all surviving adults are immune. Unaffected peripheral populations, however, may be devastated by the same diseases later on. If these populations are large enough, then these diseases soon become endemic, going through periods of high mortality, and under the right geopolitical conditions, these diseases can cause pandemics (plagues), similar to the one that we are currently experiencing. History repeats itself over and over again with enemies that are as formidable as viruses (Influenza, SARS-CoV-2, Middle East Respiratory Syndrome [MERS], etc.) and bacteria (*Yersinia pestis),* most of which have a zoonotic origin in animal reservoirs [4,20,21,22,23], a topic that has been discussed in depth in recent years. These microorganisms have been slaughtering humans throughout the 5000 years of written history that are available to us [4,12,14,24,25,26]. Current science can provide us with surprising data about that the extent to which microorganisms shape our history, but it seems that they can even program us, as suggested by recent scientific studies [27,28,29,30], giving us clues about their tremendously profound effect, which we can think of as being constant. We discuss this subject in Section 4 “Can microorganisms structure population genetics?” Traditionally, we consider microorganisms our enemies, and millions of deaths and endless suffering seem to corroborate this; hence, “villains”. However, these “micro-enemies” are more than that. There is well-founded evidence that microorganisms (viruses, in fact) have built our genomes in the same way as one may complete a puzzle that is made of pieces that have been supplied to us throughout evolution, even becoming a functional part of it in the same was as endogenous retroviruses have. In addition, our intestines are colonized by millions of bacteria, known as the gut microbiota, which perform functions that are essential for our survival. Moreover, humans have been using the fermentation process for nearly 10,000 years, and the impressive biotechnological revolution that we are currently enjoying is also possible thanks to microorganisms. In this text, we discuss the following “friendly” roles of microorganisms:

- 3.1. Microbiota, Our Bacterial Friends.
- 3.2. Endogenous Retroviruses in Human Genome: Between Disease and Evolutionary Symbiosis.
- 3.3. Microorganisms Helping Us Even before We Were Aware of Their Existence. Biotechnological Allies for 10,000 years.

Unfortunately, we are usually aware of these little “villains” as the germs that have caused millions of deaths throughout our history, but we forget the others, which are either harmless or are even beneficial to us. In this review, we will discuss the role of microorganisms as “villains or friends” and their influence on our history and on ourselves.

## 2. Microorganisms as “Villains” and Some of the Suffering They Have Caused to Humanity throughout History

The term or concept of *plague* (from the Latin *plaga,* meaning to strike or wound) and its synonym *pestilence* appear repeatedly in the Bible. This concept refers to a divine judgment as punishment for sin that takes place in the form of a virulent disease or a catastrophe that is produced through the unusual actions of natural forces, such as the 10 plagues that fell on Egypt (Exodus 9:4) [31], a veritable display of divine power in its most vengeful form. In other words, plagues are understood as unpredictable calamities and include epidemics; take, for example, this terrible and frankly disturbing fragment from the Book of Revelation in the Bible (Revelations 6:8): “*And I looked, and behold, an ashen horse; he who sat on it had the name Death; and Hades followed him. Authority was given to them over a fourth of the earth, to kill with the sword and with famine and with pestilence and by the wild beasts of the earth”* [32]. If we analyze the history of real plagues, the great pandemics, which in the light of our current knowledge are no expression of divine power, this text seems almost a prophecy. However, when we refer to *plague* in this text, we will be referring to disease.

An epidemic is a disease with a much higher incidence than expected in a specific population, and we call it a pandemic when it extends beyond the limits of a wide geographical area, such as a continent or even to the whole planet. Examples of pandemics are the influenza pandemic (Spanish flu) in 1918 and the current SARS-CoV-2 (COVID-19) pandemic. Epidemics are products of civilization, whose origins date back to the appearance of the first cities and the agglomeration of people about 5000 years ago, as deduced from texts of the now extinct Sumerian and Hittite civilizations [33]. In addition, they are the product of the increase in the population and its mass mobility through commercial routes (e.g., the Silk Road), due to migration caused by natural disasters or wars, by the armies themselves during invasions, or even when the soldiers return to their homes (one of the causes of the planet-wide spread of the Spanish influenza pandemic of 1918 [34]). However, there are many more causes. When an epidemic disease reaches a location where the population has not previously been exposed to it and, therefore, their immune systems are not prepared to deal with it, epidemics occur on virgin soil or terrain. This is what happened after the European colonial expansion in America from the end of the 15th century onwards [35], where diseases that were well known in Europe but that were absent in the New World wiped out indigenous populations to the point of near extinction and brought down entire nations and empires. This sad episode illustrates the tremendous dependence of the history of human civilization on these great pandemics.

Indeed, major epidemics do not seem possible in pre-urban tribal societies that did not have a sufficient number of susceptible people to support them. In these tribal societies, epidemics either annihilate all of the members or make them immune; however, without the proper means of transportation, they cannot infect anyone beyond the society’s natural geographic limits, thus ending the epidemic. Current science, through sophisticated molecular biology techniques, has allowed us to deduce the existence of certain infectious agents in prehistoric times, such as the hepatitis B virus (HBV), which has been found in the dental pulp of Neolithic skeletons that are up to 7000 years old [13]. Similar technologies have made it possible to detect the bacterium *Yersinia pestis* in samples from the Bronze Age that are almost 4000 years old [4,11] and even older [12,14], suggesting that the agents that are able to cause large-scale pandemics have been with humans since our origins.

### 2.1. Pandemics Have Weakened Empires. The First Records of Major Pandemics

The oldest record of an epidemic is provided by Thucydides, who cited the plague of Athens in 430 BC in his history of the Peloponnesian War [6]. Thucydides comments on the development of the epidemic and analyzes its consequences for the society and morals of the Athenians, which became weakened, heralding the end of this Athenian golden age. He also describes its symptoms, which were similar to the exanthematic typhus that is caused by *Rickettsia prowazekii*, an infection that is transmitted by the human body louse (*Pediculus humanus corporis*), which often causes epidemics following wars and natural disasters. For example, this agent was isolated along with *Bartonella quintana* in the corpses of Napoleonic soldiers after the retreat from Russia. It is the causal agent of the disease known as Trench Fever [36]. Similar cases were also reported among combatants during the two world wars.

In the 2nd century AD in the Roman Empire during the reign of emperor Marcus Aurelius, Galen described another plague, known as the Antonine plague, that lasted from 165 to 170 and comprised a hemorrhagic rash and presumably a fever that may correspond to smallpox, which is caused by the variola virus. This plague, which has been linked to the decline of the Roman Empire and the rise of Christianity, was carried by Roman troops upon their return from the war with the Parthians in Mesopotamia. This epidemic may have been the cause of death of the emperor Lucio Vero of the Antonina family in 169 AD, hence the name of the epidemic. The reappearance of the epidemic 9 years later caused the deaths of some five million people throughout the empire, decimated the Roman army, and weakened the empire [7].

In the 6th century AD, the Byzantine historian Procopius of Caesarea described the so-called “Plague of Justinian” [8,37,38], which is considered to be the first known pandemic in the history of mankind. The bacterium *Yersinia pestis*, the causal agent of the plague, has been isolated from ancient archaeological samples, indicating that this terrible disease probably originated 5000 years ago in the plains of Central Asia, somewhere between modern China, Mongolia, Russia, and Kazakhstan [12,14]. In this regard, there are biblical references to a possible plague epidemic among the Philistines of Ashod (11th century BC) [39,40], and it has even been speculated that this disease could be related to the weakening of the Sumerian Empire more than 4000 years ago (18th century BC) [33]. However, the Justinian Plague (541–542 AD) that is described by Procopius is still considered to be the first pandemic in human history. This pandemic began in Egypt and Ethiopia, and expanded rapidly and had terrible effects, causing an estimated 20 million deaths in the Mediterranean Basin alone [37,38]. The symptoms that were described by Procopius, swelling or buboes (swollen lymph nodes) in the armpits and groin and their transmission by rat fleas (*Xenopsylla cheopis*), seem to correspond to the epidemic that was transmitted by *Yersinia pestis*. This agent was identified in a paleo-microbiological study that was carried out in 2012 on the dental pulp of skeletons that were found in a Bavarian necropolis in Aschheim near Munich that were dated to between 500 and 700 AD [41]. The Justinian Plague seems to have started in Egypt in 541 AD, but its possible origins were the great lakes region of West Africa*,* where, in turn, it would have arrived from its much older Asian niches [4,11]. The epidemic reached Byzantium in 542, during the Justinian reign and was probably introduced by the troops of Belisarius during his conquest of Egypt (again, soldiers returning to their homes). The pandemic appears in Arles in 549 and is mentioned by Bishop Gregory of Tours (539–594), spreading throughout Europe through multiple outbreaks. According to the Byzantine historian Procopius, this plague “*was so contagious that it almost took all of humanity*” [8], with an estimated mortality of up to 50% of the European population. However, more recent studies [42] have questioned this impact at the continental level, indicating that it may have had more relevant effects at the local level, such as on Byzantium itself. In fact, the authors of that time who commented on this plague did not devote much attention to it in their writings: Procopius and Gregorio de Tours dedicated around 1% of their writings to this plague [42]. However, it seems that large regions of the Byzantine empire were depopulated; in the city of Byzantium alone, almost 300,000 people, which accounts for approximately half of its population, died [37,43]. Today, we know that this infection has a mortality rate of 50% without treatment in its bubonic form. In its pneumonic form (5%–15% of cases), after an incubation period of 2–3 days, it presents with a very high fever, tachycardia and a cough with bloody sputum, and a mortality in excess of 90%. Additionally, the septicemic form, which can be associated with both forms, is the most sudden form, causing death without giving time for the typical manifestations of the disease to appear [37,43]. This pandemic had tremendous consequences for the future of the Byzantine empire and probably promoted the rise and consolidation of the Islamic people that conquered more than half of the empire. In this way, the demographic weakening of the vast Byzantine Empire facilitated the Arab-Muslim conquest a century later and the changes in maritime trade from the Mediterranean to the North Sea [37,43]. Likewise, the Slavic invasions of the 6th century were greatly favored by these demographic losses, dashing Justinian’s plans to recreate the Roman Empire of Constantine, including reconquering Italy, which was limited to Ravenna [37,43]. The epidemic radically changed the social reality of Greece and Eastern Europe through the definitive settlement of Slavic peoples [43]. In addition to the tremendous political and social changes that were created by this disease, this epidemic led to important preventive measures such as the draining of swampy areas, where the *Anopheles* mosquito, the variety that causes malaria, nested, resulting it in being eradicated due to this draining process and due to the clearing of the forests and the felling of trees to obtain wood for construction using a weapon of war: the axe [43].

### 2.2. The “Black Death”, the Great Plague Pandemic of the 14th Century. The World Changes at the Huge Cost of Human Lives

**Figure 1:**
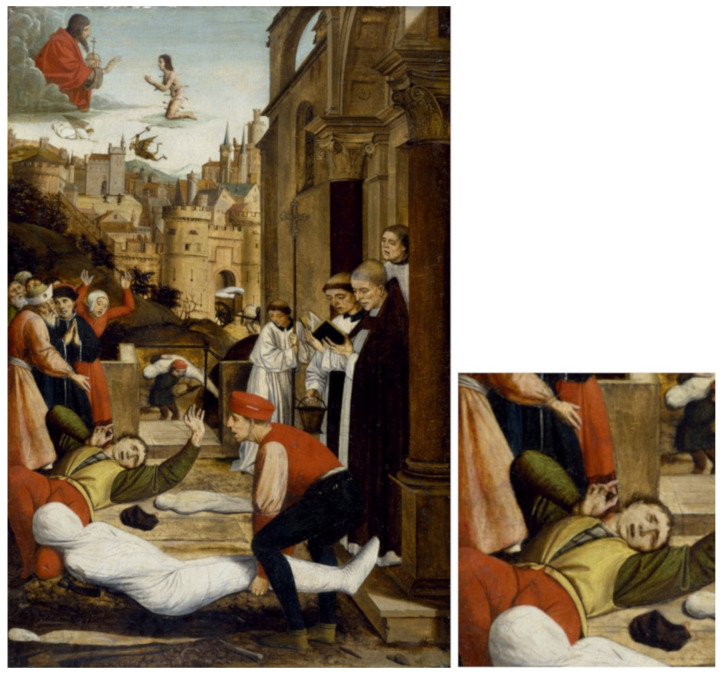
Adapted from “Saint Sebastian interceding for the Plague Stricken in Pavia”, created by Josse Lieferinxe (French painter, active 1493–1505). Source: The Walters Art Museum, Baltimore [51].

In the mid-fourteenth century, starting in 1347, the most terrible and possibly best-known episode of the great pandemics of history began: the Black Death, which has also been called “The Plague” and “that evil that spreads terror”, which broke out periodically [10,44,45], as it had already done in the 6th century in Byzantium; the disease presented itself with the same symptoms as those observed by Procopius during the Byzantium epidemic: large buboes in the armpits and groin. As explained above, the bacterium *Yersinia pestis* is the causal agent of plague, a disease that probably originated 5000 years ago in the plains of Central Asia [12,14]. The name “black death” seems to come from a misunderstanding when translating the Latin expression *atra mors,* where *atra* can means both “terrible” and “black”. This “Black Plague” has been well described by the chroniclers of the time, such as Boccaccio (in his *Decameron*) in Italy or Jean de Venette in France [46]. In the present, this plague has been extensively documented by Ole J Benedictow [10] and in the collection of documents reported by J Alberth [47], which includes a letter from 27 April 1348 by the musician Lodewijk Heyligen (Ludovicus Sanctus), and there are additional interesting documents in [48] that describe the apocalyptic development of pandemics. The Sienese chronicler Agnolo di Tura wrote of his own terrible experience: *“There are not words to describe how horrible these events have been… [I] have buried five of my sons with my own hands”*, adding, “*no one weeps for any of the dead, for instead everyone awaits their own impending death”* [49]. This catastrophe was even witnessed in art [50] and can be seen in such works as “The Triumph of Death”, fresco from the Pisa cemetery, Francesco Traini or Buonamico Buffalmacco, 1350 and Burying the victims of the Plague, an illustration from the manuscript of Guilles de Mussis, Annals of the Plague in Tournai 1349, MS 13076-77, f 24v. Figure 1, which shows Saint Sebastian interceding for the Plague Stricken in Pavia [51] is an example of that influence. In that painting, the bubo on the neck of the figure in the lower left can be observed (detail). Additionally, there are other illustrations that have been said to be related to this Plague, such as an illustration of Plague victims with buboes that is present in the Toggenburg Bible 1411 [50].

**Figure 2:**
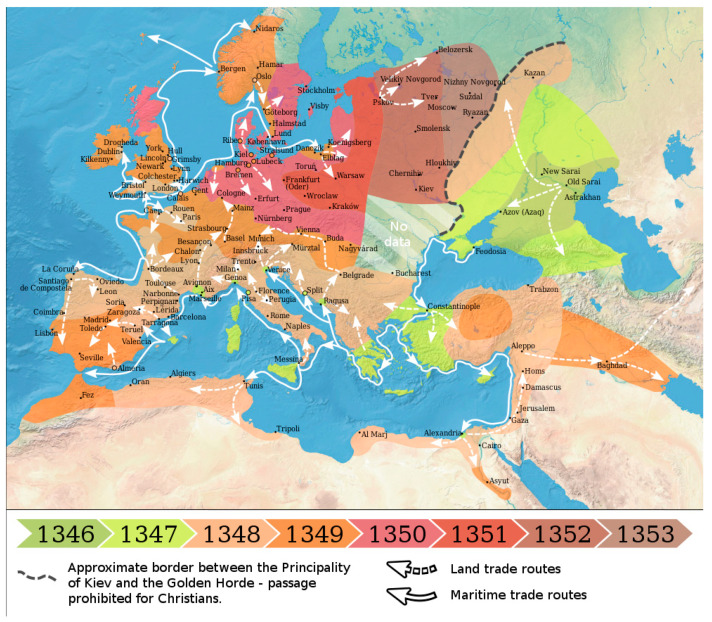
Spread of the Black Death in Europe and the Near East (1346–1353). The contagion route can be easily followed from East to West: In the summer, the Black Death arrived in Constantinople; in September, it arrived in Alexandria; by October, it was already in Sicily, and in January 1348, it arrived in Venice, Genoa, and Marseille. It reached Barcelona in May, Valencia and Almería in June [43], and upon reaching the British Isles and the Scandinavian Peninsula, it had covered Europe from end to end. Source: By Flappiefh—Own work from Natural Earth; The origin and early spread of the Black Death in Italy: first evidence of Plague victims from 14th-century Liguria (northern Italy) maps by O.J. Benedictow., CC BY-SA 4.0, https://commons.wikimedia.org/w/index.php?curid=66468361, accessed on 27 July 2021.

The Plague started in Asia, crossed Persia and the Middle East, and then entered Europe, which, according to the data compiled by Benedictow [10], would have killed 60% of the European population, which, when considering that there were between 50 to 80 million inhabitants of Europe at that time, represents a brutal catastrophe. This terrible pandemic reduced the world population from an estimated total of 450 million to around 350 million. For example, England’s population decreased by almost 70%, falling from about six million inhabitants before the Plague to only about 2.5 million afterwards [52]. London may have lost two-thirds of its population during the outbreak of 1348–1349. The spread of the Plague through Europe has been reviewed by Benedictow [10], who describes how fleas and their terrifying bacterial occupant probably travelled in the caravans and boats nestled comfortably in the hair of rats from China via the Silk Road, where merchants did not notify the people they came into contact with of the first cases to preserve economic activity, allowing the disease to once again spread throughout Europe, first through the Mediterranean basin and later through Atlantic Europe. Data from that period also indicate airborne contagion. Thus, the Plague of 1348 presented two versions, each one more terrible for regions with large populations and related activities: the bubonic, blood-borne form, and the pneumonic form, which is borne in the air. This terrifying disease spread throughout Europe and reached Scandinavia within months (Figure 2).

According to Benedictow [10], the movement of the Plague followed two main routes: over land in a south-westerly direction and by ship through the Black Sea towards Western Europe, which caused the greatest damage, and along the Volga River and part of the Don River, where the Plague was already reported in the spring of 1346. It is very likely that the Mongols and their caravans inadvertently transported the Plague from Central Asia to the Eastern European cities of Constantinople and Trabzon in 1344. Pandemics usually move from East to West, and it is in this direction that the pandemic that devastated Europe is assumed to have moved, from the territories of the Golden Horde, a central Asian Mongolian state that encompassed part of today’s Russia, Ukraine, and Kazakhstan, thus including the region where *Yersinia pestis* has recently been detected in archaeological samples from the Bronze Age [11]. Trade caravan routes from China to Crimea had been established as important links through the great Mongol empire founded by Genghis Khan in the 13th century, whose grandson would seize southern Russia between 1236–1240. Thus, the Mongol conquests united China, Central Asia, Persia, and southern Russia, including Crimea, in an immense empire, which allowed the existence of trade routes to Europe. This empire was split into several states upon the death of Genghis Khan, the so-called “Hordes” [10].

There is a description of a serious epidemic in Azerbaijan in 1346–1347 [53], but the Plague devasted the territory of the Golden Horde in October and November of 1346, including its capital Sarai, and large regions of this empire, which was “referred to as the country of the Uzbeks” [10]. The epidemic reached Trabzon, a major Black Sea trading settlement, and was probably carried by refugees from both Caffa and other parts of the Caucasus who had become infected in the Volga and Don basins. The disease reached the border regions of Asia Minor, Mesopotamia (present-day Iraq), and Persia (present-day Iran) in 1347, and in 1349, it struck the great plain of Anatolia, an area that was occupied by Muslim Turks. The Plague would reach Alexandria in the same year of 1347, in the Mamluk empire of Egypt (which included Palestine), and its arrival was probably due to trade between this empire and the Mongol Golden Horde. From Alexandria, the epidemic would spread through the Nile valley, reaching Aswan in 1349 and spreading through North Africa by the usual vectors: merchants, refugees, and pilgrims. In the Middle East, mortality was particularly high in rural areas, including in large areas of Palestine and Syria, reaching total depopulation in some provinces. In Palestinian lands, the Plague reached Gaza in 1348. In Syria, 400,000 people died, more than 100,000 people died in Aleppo, and in Damascus, more than 30% of the population was eliminated. In Muslim lands, the rite of resorting to divinity to explain the epidemic was followed, which was also the case Christian lands, with assumptions being made that the epidemic was a divine punishment for the sins of men [10,43].

In 1266, the Golden Horde allowed the settlement of the Genoese (Italian) trade consulates in Crimea, such as Caffa, but the Khanate of Kipchak (belonging to the Golden Horde) converted to Islam, including its Tatar populations, generating the rejection of Christian merchants. In 1343 the Khan of Kipchak, the sovereign of the Golden Horde, ordered an attack on Italian trade settlements such as Caffa, many of which were able to resist the siege. The Tatar soldiers fell ill with the Black Plague that would have already appeared in the territories of the Golden Horde and decimated the Mongol army. The Khan of Kipchak ordered an end to the siege, but before leaving, the Tatars catapulted the corpses of the Plague victims into the city, a true act of biological warfare. However, although it is evident that the Plague infected the city of Caffa, this was probably not due to the infected Tatar corpses. Today, we know that people who are killed by the Plague are not contagious, with the exception of the presence of fleas on the clothing on their corpses, which is strange since fleas abandon the bodies of the dead as soon as they notice a decrease in their temperature. It is much more likely that rats entered the city during the siege because while walls may prevent besiegers from entering, they are no barrier to rodents [10,43].

Some Genoese merchants who had fled from Caffa in their ships, docked at the port of Messina in Sicily in early October 1347. In July 1347, the Plague arrived in Constantinople, one of the few metropolises that existed at that time; those who fled the city due to the disease would disperse themselves throughout the rest of the Byzantine territory [10]. From Constantinople, the Plague spread throughout the rest of Europe via trade routes (Figure 2). This plague returned many times during the 14th and 15th centuries, with infections being reported in 1362, 1368, 1374, 1381, 1390, 1399, 1405, 1410, 1423, and 1429 [49].

In addition to the presence of *Yersinia pestis*, other factors conspired to create the perfect storm that was the Black Death. Before the Plague, Europe had already been devastated by war, famine, earthquakes, and other disasters. For example, an unusual climate in Europe allowed the fleas to thrive. This climate also damaged crops and caused famines, with a shortage of fruits and vegetables that weakened the immune systems of Europeans, compromising their ability to cope with the disease [54]. Another of these disasters—war—is believed to have been the principal route through which the Plague first made its way to Europe. Death was so extensive that there were frequently not enough clergy to administer last rites to the dying, and the clergy were hit harder than the average population because of the close living quarters that are common to convents and monasteries. It took a century for the European population to recover, but this huge demographic reduction that was caused by the Plague was the driver of all the changes that happened afterwards [55], as we will discuss later.

The continual recurrence of the Plague throughout the years, every six years on average, as previously mentioned, forced medieval populations to accept the Plague as a part of life. Humanity was forced to live under a sword of Damocles, with the constant threat of widespread sudden death. However, despite its virulence, each new outbreak finally ended for two reasons: first, with such high mortality, there were not enough hosts for *Yersinia pestis* to survive [10]. In addition, acquired immunity limited new diffusion, which can be deduced by an interesting comment of the emperor of Constantinople, John VI Kantakouzenos, who stated that *“the few who recovered did not suffer a second attack, or at least not of a serious nature”* [56]. This peculiar emperor who occupied the throne in 1341 was also a historian and theologian and wrote four books of memoirs, which contain the history of the Greek empire from 1320 to 1360.

In relation to the possible transmission pathway, the ancient physicians Hippocrates (460–370 BC) and Galen (129–210 AD) championed the theory of “miasma”, or poisoned air, to explain the transmission of diseases, which medieval Europeans believed to have caused the Black Plague. In contrast to the miasma theory and even astrological or religious explanations, a doctor from the Muslim kingdom of Granada in Spain had already affirmed the possibility of contagion between people, stating that “*the best we learn from our extensive experience is that if someone comes into contact with a sick person, they are immediately affected by the same disease, with identical symptoms”* [54]. People of that period thought that hot baths allowed the Plague miasma to enter humans, so the public baths were closed. The clothes and belongings of the victims were thought to be contaminated and were burned, and cats were killed as possible transmission agents. The so-called Plague doctors wore protective clothing with a long cape, a mask, and a beak-shaped part over the mouth and nose that contained aromatic substances (partly to block the putrid odor of decomposing corpses), perhaps an early version of today’s modern hazmat suit.

#### 2.2.1. Did the Plague Only Travel to Western Europe?

In general, the effects of the Plague in Asia are less thoroughly documented than those that occurred in Europe. The effects of the Plague in Asia are based on both population figures during that period and on estimates of the number of victims of the disease in population centers. In the Chinese province of Hubei, up to 80 percent of the population died [43]. As a result of famines and plagues, China’s population went from 125 to 65 million between 1200 and 1350 [57], although this enormous decline cannot be solely attributed to the Plague. In addition to commercial relations as a vehicle for spreading the pandemic, war once again contributed to this disastrous situation. During a siege of Bagdad, as in Caffa, the army was infected with the Plague, and it entered the city; remember that walls are not an obstacle for the rats and their fleas. Arabia may have been infected by pilgrims to Mecca who carried the Plague. This represented a religious problem since Muhammad had promised that the holy cities of Medina and Mecca would not be attacked by epidemics. In fact, Medina did not suffer the epidemic, fulfilling this promise, and the fact that Mecca was affected was attributed to “the presence of infidels” [10].

The last great Plague outbreak began in Yunnan province in China, halfway between the 19th and 20th centuries (1885–1920), and spread to several countries, remaining active until 1959. In China, the areas of Manchuria, Mongolia, and central Asia accounted for the highest number of deaths—about 12 million [43]. In 1894, during this last great episode, the Swiss scientist Alexandre Yersin identified the bacterium *Yersinia pestis* as the causal agent of the disease [58]. Today, the Plague remains endemic in several parts of the planet, and in the last 20 years, Plague epidemics have occurred in South America, Asia, and especially Africa, mainly in small towns and villages or agricultural areas [59].

#### 2.2.2. How Did the Black Death Change History?

This medieval pandemic once again altered the history of humanity, and for the survivors of the Plague, the aftermath was better than expected [10]. Before the pandemic, there had been an increase in the prices of essential foodstuffs and a downward trend in income due to population growth, which limited access to agricultural resources. However, all of this changed suddenly. The scarce labor force increased the relative demand for work, pushing up wages. Nobles, wealthy peasants, and urban employers had to compete for workers. The owners of the means of production saw their profits fall through, finding themselves having to pay much higher wages and forcing them climb down the social ladder, while ordinary people saw a very substantial improvement in terms of their material well-being. For example, the wages of construction workers in the 15th century were comparable to those of the 19th century and were even higher than those during the First World War [60]. The ”golden age of bacteria” resulted in the ”golden age of salaried workers” [10]. Abandoned fields were reforested or converted into pasture for livestock, which required less labor than growing cereals, thus increasing the consumption of meat and dairy products such as butter, which enriched the average diet of ordinary people. The level of consumption of the working classes increased, reactivating the economy. Consumer society took its first steps. The centers of the economy shifted to regions producing consumer goods, fabrics, footwear, etc., from Italy to the Netherlands and England, which would emerge as new commercial powers. The use and design of new tools was improved to facilitate work, such as water-powered sawmills. In practice, serfdom disappeared in many regions of Western Europe, and relationships between peasants, artisan workers, and their lords or employers increasingly took on the characteristics of a mercantile system, in which the levels of income and wages were determined by the supply of and demand for labor. In contracts between nobles and peasants, which were in the possession of both parties, feudalism was terminated. For similar reasons, kings, princes, city states, and urban governments improved and professionalized their administrations, seeking to decrease costs and to increase fiscal efficiency, improving public service benefits. Large ecclesiastical landowners, monasteries and convents, magnates, aristocrats, and even petit bourgeois landowners found it difficult to hire laborers at such high wages, resulting in a significant decrease in their income. A possible solution for these potentates was war. At that time, one of the longest wars in history was underway between France and England, the 100 Years War, which started in 1337 and lasted until 1453. Kings and rural owners perhaps found a way to continue collecting war fees to maintain and prolong warfare as well as to take advantage of the spoils and thus maintain their lifestyles and social prestige [10]. In Spain, the financially distressed aristocracy participated in the wars against the Muslims of the Nasrid kingdom of Granada, which was finally conquered in 1492, with more determination, stimulating the funding of Christopher Columbus’ project to open up a new western sea route to India as an alternative to the Silk Road, which was complicated and dangerous in those times, as the existing routes crossed through Islamic states [10].

**Figure 3:**
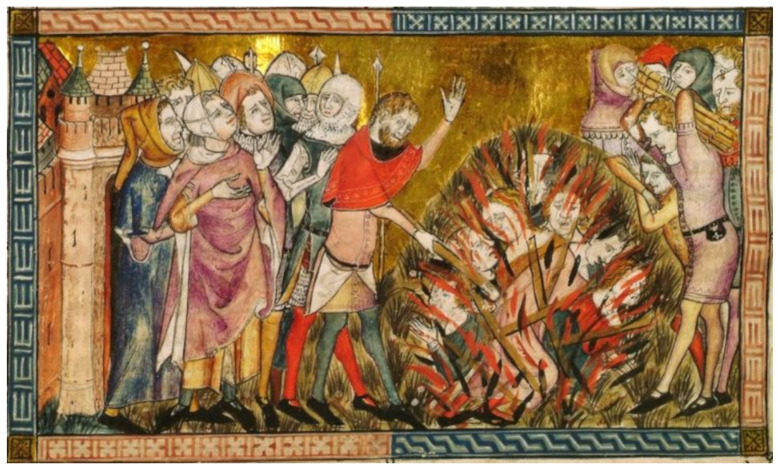
Jews being burned at the stake in 1349. Miniature from a 14th century manuscript *Aniquitates Flandriae* created by Pierart dou Tielt (Belgian miniaturiste, 1340–1360). Source: By Pierart dou Tielt (fl. 1340–1360)-http://balat.kikirpa.be/photo.php?path=X004175&objnr=20049662, accessed on: 27 July 2021. Public Domain, https://commons.wikimedia.org/w/index.php?curid=64384805, accessed on: 27 July 2021.

The Plague generated religious panic; large groups of flagellants roamed the cities stirring up anti-Jewish sentiment. The fact that Jews had a lower mortality than Christians in some communities was interpreted as proof that they were responsible for the pandemic, giving rise to multiple pogroms, such as that of Barcelona on 17 May 1348 despite the intervention of King Peter III of Catalonia, whose second wife, Leonor, had died of the disease in the same year [61]. This sentiment led to real genocides against Jews throughout Europe, who were blamed for causing the disease by “poisoning wells and other sources of drinking water” (Figure 3). These persecutions could not be avoided, even with the intervention of the pope, who published two bulls against them and even condemned the flagellants [10]. Interestingly, we now know that carriers of recessive familial Mediterranean fever (FMF) mutations have natural immunity against *Yersinia pestis*, and this hereditary disease is especially frequent in Jewish populations (Sephardim of Spanish origin, Ashkenazi, Central European and Mizrahi), as will be commented upon in detail in Section 4. Can microorganisms structure population genetics? This may explain why Jews died from the Plague at lower rates than Christians [62]. Despite these episodes, one of the most important historical changes that was caused by the Plague was that religious explanations for everything, regardless of specific faith—good or bad harvests, epidemics, good or bad luck, etc.—slowly shifted to rationality and to the formulation of scientific explanations and approaches. A greater understanding of the ways in which epidemics can spread was achieved, starting with epidemiology. Health committees were created to combat the disease, assuming that the administrations had a responsibility to protect the health of the citizens.

The Plague continued to be a scourge of humanity between the 16th and 19th centuries, causing major outbreaks in Europe, resulting numerous deaths. In 1628, it caused 20,000 deaths in Lyon, and in 1630, it was responsible for a million victims in Lombardy, representing 63% of the region’s population. In 1649, the Plague killed 20,000 people in Seville and 20,000 in 1651 in Barcelona, half of the city’s population. In 1656, the Plague returned to Italy, causing 250,000 deaths in Naples, half of the city’s inhabitants (reflected in Garguilo’s painting, “The market square of Naples during the Plague”). Naples was followed by Venice, Padua, and Verona, with mortalities accounting for up to 70% of the population. In 1665 in London (England), it killed 100,000 people (in addition to the city’s great fire of 1666). In 1679, the Plague claimed more than 70,000 victims in Vienna as well as in North Africa, America, and Asia, where terrifying figures were observed once again and that were very similar to those from outbreaks in the 14th century [10,43]. What changed after this new microbial catastrophe? According to data compiled by Benedictow [10], Trinity College, Cambridge University, closed its doors due to the Plague epidemic in 1665. The 23-year-old Isaac Newton was studying there and had to return to his home in the nearby village of Woolsthorpe. During the two years of confinement, Newton wrote his famous *Philosophiae Naturalis Principia Mathematica*, one of the most pivotal works in history. One may wonder whether he would have made these discoveries if he had he stayed at Cambridge. Perhaps the forced stay in Woolsthorpe Manor, where he may have had fewer distractions than he would have at Cambridge, favored him devoting all of his energy to revolutionizing physics. We will never know. In any case, he made good use of his confinement. However, many more things happened that are closely linked to this disastrous pandemic. Health took on considerable importance; in England, it was understood that it was an obligation of the state, and something similar happened in other countries, leading many hospitals being built. Even in everyday life, this principle was applied through the use of toilet paper, the use of soap, sewer systems, city pavement, trees and gardens, etc. [10]. Perhaps this pandemic contributed to triggering major historical revolutions: Due to the scarcity of manual labor, people became accustomed to the gradual mechanization of their jobs. It was in this context that the steam engine appeared and the industrial revolution and other more social revolutions began, such as the so-called Glorious Revolution in England in 1688, where a Parliament that would control the power of the king was elected [10]. Later, in 1789, the French Revolution arrived after a late episode of the Plague in Marseille in 1720 that caused almost 100,000 deaths. In medicine, superstitions began to disappear. In 1767, James Lind linked scurvy to vitamin C, and a great revolution took place in 1796, when Edward Jenner created a vaccine for smallpox, which had killed millions of people and had almost extinguished the poor inhabitants of the Spanish colonies in America. In the 19th century in London, John Snow, who is practically the father of epidemiology, discovered that cholera, another historical scourge, was transmitted by water that was contaminated with fecal matter. Later, Robert Koch would describe the bacterium *Vibrio cholerae* as the causal agent of cholera [10,43]. There is thus sufficient data for us to think that this new traumatic experience of the Plague acted as a trigger for tremendous changes, including many positive ones.

#### 2.2.3. The Plague, a Scourge Much Older Than We Thought? A Terrible Traveling Companion for Millennia

The early Bronze Age was a time of change. People in Europe began smelting copper and tin together to make bronze items and weapons, and herding intensified, with the herding of cattle, goats, and sheep becoming increasingly important. The first cities with tens of thousands of residents, such as Uruk in present-day Iraq, arose around 2900 BC. Thus, urban agglomerations, sedentary lifestyles, and the chances of contact between humans increased; trade with other communities was invented, substantially increase the likelihood that germs would be exchanges. The first cities would become breeding grounds for pathogens, and sedentary behavior and the appearance of agriculture and livestock intensified contact with domestic animals, providing the ideal conditions for microbes to jump from animals to people. Perhaps the herders themselves developed a tolerance to the diseases of the animals that they cared for and even lived with, but they could see that these microbes could be carried to urban populations who were naïve to these infections, where they would be lethal to residents, similar to what would happen millennia later, in the 16th century AD, with the conquest of America by the Spanish.

There are startling changes that were observed in the human population at the beginning of the Bronze Age. The study of ancient DNA samples has revealed a mix of early farmers and hunter-gatherers living during the Neolithic Period (or New Stone Age) in Europe. Then, when the Bronze Age began, there was an infusion of new DNA. It appears that three-quarters of the genetic makeup of people from the early Bronze Age flowed from eastern steppe areas, such as present-day Russia [63]. Thus, Europe changed dramatically during the Bronze Age, with large population changes generally being attributed to the rise of new metal technologies, trade, and climate change. However, another reason for this social upheaval cannot be ruled out: major blights, such as the Plague itself, was possibly carried by newly domesticated horses, as these new means of transport allowed for long-distance travel and the transport of germs to distant human communities and places. However, conjectures aside, do we have real data on the presence of the terrible *Yersinia pestis* in such ancient times? It seems that we do. Recent genomic investigations with samples from the late Bronze Age from around 4000 BC [11] have identified that *Yersinia pestis* variants had already adapted to the fleas that were present in Eurasia (Samara region in modern-day Russia), and strains that had also adapted to fleas from 2900 BC have been detected in Armenia [14]. Despite the antiquity of these samples, there is evidence of the presence of *Yersinia pestis* in Eurasia from even earlier, as previously discussed. For instance, a recent study by Susat et al. [12] reported the reconstruction of the genome of a 5000-year-old *Yersinia pestis* strain from the remains of an adult hunter-gatherer from Latvia. However, these ancient variants seem incompatible with adaptation to arthropods, so it can be deduced that they would not be capable of being transmitted by fleas [11,14], and an alternative transmission mechanism cannot be ruled out [4,64]. To analyze the diffusion of these strains among humans from this remote time, it must be taken into account that the analysis of the human genome from samples from the period between 5500 and 3200 BC demonstrates that the Central Asian steppe region played an important role as a migratory corridor throughout the Bronze Age, probably facilitating the spread of human-associated pathogens across Eurasia. For example, the migrations of shepherds of the Yamna culture, which was located in present-day Ukraine, ranged from the Pontic-Caspian steppe (between the Black Sea and the Caspian Sea) to the west, to Europe, and to the east in Central Asia and the region from Altai, which borders Mongolia. These migrations resulted in mixing with local Neolithic farmer populations, forming the gene pool that appears to constitute European populations to this day [65,66,67].

These findings seem to lend credibility to a possible Plague epidemic more than 4000 years ago (18th century BC) in Sumer, present-day Iraq, that catastrophically affected the population and that could perhaps be linked to the disappearance of what was the first known civilization in around 1950 BC, as reported by Amjad Daoud Niazi [33]. This study was based on the translation and interpretation of clay tablets that were written in cuneiform that relate what are known as the “Lamentations”, which were translated in 1940 by the great Assyriology expert, Samuel N. Kramer [68]. The “Lamentations over the destruction of Ur” [68] refers to an extended period of drought and desertification that practically destroyed this city, which was the center of one of the so-called “irrigated states”. This desertification was accompanied by a terrible famine and consequent mortality. While these tablets do not explicitly mention the disease as we know it now, they include passages that can be taken as signs and symptoms of it, suggesting a pandemic, which was also reported by Niazi when interpreting these cuneiform texts [33]. These texts tell of an invasion of the neighboring Elamites that were east of Sumer from the Zagros Mountains and comment on a possible enzootic source in this region. As always, the mass movement of people, such as in wars with attacking troops and fleeing refugees, favors the transport of the disease vectors: rats and fleas. The walled cities thus constituted a form of isolation for the Sumerian population that would have hindered previous contact with this possible Plague and that caused many inhabitants to be susceptible to the infection, both of which are optimal conditions for an intense spread of the disease, the results of which being a pandemic that was devastating in its weakening of a millennial empire that would be all the more severe if airborne transmission was also present, as this text of the so-called “Lament of Sumer and Urim” seems to indicate, stating that “*the people, in their fear, breathed only with difficulty. The storm immobilized them, the storm did not let them return*”, where storm could indicate the disease that immobilizes them and has no solution and that also causes respiratory problems. It is also stated that the highest deity in Sumer (Enlil) “*sent down Gutium from the mountains. Their advance was as the flood* (…) *The great wind of the countryside filled the countryside* (…) *the extensive countryside was destroyed* (…) *afflicted the city with that which cannot be withstood with weapons* (…) *they gasped for breath* (…) *how long until we are finished of catastrophe* (…) *Inside Urim there is death, outside there is death*”. The *Gutium* were Elamite people who may have been the bearers of the Plague that could not be stopped with weapons, suggesting that it was not a destruction that was caused exclusively by war because weapons were unable to resist it. The great wind or the storm may refer symbolically to the disease. Other texts talk about “lethargy” that was possibly caused by fever and buboes and respiratory difficulties. Multiple affected cities are cited: Ur, Urig, Nibru, and Eridug. From the “Lament of Eridug”: “*Along with the fluids spilled from his guts, his blood spilled forth*”. This last sentence may be a reference to yellow fever, but there is no additional data to indicate this possible infection. As such, texts from different cities of the Sumerian empire seem to coincide, suggesting a pandemic that arrived from the East with the invaders that came from there, where it may have been endemic in fleas, and do not appear to refer to the destruction caused by droughts and desertification or war, although it is likely that war was the trigger for the pandemic, as had been the case for millennia. The possible pandemic of the Sumer empire may be the first chronicle of the Plague, although recent studies that have been discussed in this section [4,11,14] have shown that the DNA of the Plague can be detected in the pulp of the teeth of Bronze Age skeletons, with up to 8% harboring what was most likely the bacteria that caused their death.

Other pandemics from antiquity have been suggested following the analysis of archaeological remains, which brought, similar to the Plague, tremendous consequences for well-established empires, once again shaping the history of humans. This may be the case of the great epidemic that ravaged the Hittite empire [69] for several decades. Thus, in the Hittite civilization, which was located in the interior of Anatolia, at the time of King Mursili II (1321–1295 BC), a terrifying epidemic occurred that devastated cities and fields. The fact that it lasted for several years (more than two decades) reflects that it was not an ordinary epidemic. Symptoms, mode of infection, and geographic area appear to identify the causative agent as *Francisella tularensis,* and it ravaged the eastern Mediterranean in the 14th century BC. It dates back to an outbreak in Canaan along the Arwad–Euphrates trade route. This same agent is credited with the outbreaks in Canaan in around 1715 BC and 1075 BC. Initially, the epidemic of the 14th century BC contaminated an area that extended from Cyprus to Iraq and from Israel to Syria, without affecting Egypt and Anatolia due to quarantine and political borders, respectively. Later, war spread the disease to central Anatolia, and it is from there that it was carried to western Anatolia, constituting the first known record of biological warfare, which would be committed 3 millennia later by the Tatar soldiers in Crimea, initiating the terrible Plague of the 14th century AD that would ravage Europe. Eventually, the Aegean soldiers who were fighting in western Anatolia returned to their islands, spreading the epidemic further.

### 2.3. America “Ceases to Be an Island for Plagues” and Almost Succumbs to the Arrival of Europeans. Culturization or Assimilation? Facilitated by Microorganisms

After the terrible plagues that devastated Europe in the Middle Ages and that had made their way to the old continent through trade routes with Asia, such as the Silk Road, it seemed necessary to explore other routes such as traveling by ship to the East by sailing West. How could this be possible? Just assuming that the world was round, something that seemed evident from the Greeks (the phases of the moon, with the curved shadow of our planet on it, the masts of the ships when moving away, etc.) had been forgotten or stigmatized in the darkness of the Middle Ages. Christopher Columbus recovered this idea, perhaps counting on some direct testimony, and he proposed the idea and came to the “Indies” thinking that it was China. However, Columbus not only brought sailors with him, he also brought other invisible stowaways, microbes, which traveled with him and with other explorers who came after him. Upon landing in the Indies, which turned out to be a new continent (the “New World”), the exported microorganisms came close to annihilating its inhabitants. These microorganisms found “virgin soil” by infecting populations that did not have immune protection, as they had not previously been in contact with them, producing tremendously virulent diseases [35]. It would not be the Spanish soldiers (Spaniards) or their sophisticated European weapons who conquered the continent; the true conquerors were diseases such as smallpox, measles, and typhus [70]. Just three decades after the arrival of the Spaniards, that “New World”, which was probably just as old as ours, almost disappeared, or more correctly, was assimilated [70]. The destructive action of the newly arrived microorganisms was added to the no less destructive actions of the Spanish colonizers themselves, as already indicated by Antonio de Herrera [70]. During the first century after the arrival of Europeans, there were multiple epidemics that were characterized by high mortality rates [70]. The major epidemic diseases that were imported to America by European colonizers between the 15th and 18th centuries are summarized in Table 1. Most of these diseases seemed unfamiliar in the pre-Columbian New World, such as measles, malaria, smallpox, influenza, epidemic typhus, chickenpox, and diphtheria. Up to 14 epidemics in Mexico and up to 17 epidemics in Peru were recorded to have occurred between 1520 and 1600 and were the main cause of the destruction of two great empires (Aztec and Inca) that existed in these regions, along with millions of people. The English and French colonizers in North America, sadly, played an analogous role to that of the Spanish and Portuguese in Central and South America, decimating additional indigenous communities (Table 1). Clearly, the deadliest of the first epidemics were those of “eruptive fevers”, especially smallpox, but also measles or typhus, which, due to the lack of specific details about each disease of that time and the superimposed nature of those epidemics, we cannot distinguish correctly.

The local populations were so devastated by both infection and prostration that they could offer little resistance to their European conquerors. Various reports of disabling epidemics with many victims strongly support the theory that these diseases decimated aboriginal populations. Hernan Cortes took advantage of this catastrophe to defeat the Mexica of the Aztec empire. It is assumed that the fall of Tenochtitlan, the capital of that empire, was more due to the work of smallpox than to any Spanish military strategy [43]. By 1531, the population of Mexico had been reduced to a third of what it had been pre-European contact. The Inca empire that extended from Peru to Bolivia for 3500 km from north to south and 800 km from east to west and had a powerful structure, followed a similar fate to the Aztecs. How can the submission of millions of Incas to just 200 men lead by Pizarro be explained? Similar to the Aztecs, the Incas would have been weakened and decimated by the diseases that had been brought over by the Spaniards and that were previously unknown to the Amerindians: smallpox, measles, influenza etc. With a population of about 10 million people in 1532, a census covering the period from 1548 to 1553 counted 8.3 million inhabitants, and in 1791, there were only one million inhabitants [70]. The easy access to the Aztec and Inca empires by ship and their high population densities allowed for the rapid and effective transmission of diseases. Just 30 years after the arrival of the Spanish, an estimated 50 million Americans had died [43]. Of the different diseases (Table 1), smallpox was extremely lethal to Native Americans but barely so to Europeans due to the lack of exposure of these native communities for millennia, while Europeans had been frequently exposed in childhood, although other factors, including genetic sensitivity, cannot be ruled out. Other infectious diseases such as rubella, typhus, malaria, and yellow fever were also disastrous [71], as they would also become for other non-American communities that would become colonized by the Europeans. Although assumed to be less virulent than smallpox, rubella caused a vast number of deaths when it was introduced into populations that were previously naïve to this infection. Thus, in the Faroe Islands (between Iceland and Norway) in 1846, 6000 of the 7800 inhabitants were infected with rubella, but only 100 died, in contrast to the islands of Fiji, where 40,000 of the 150,000 inhabitants died, suggesting an ethnic factor in the susceptibility to this infection [70]. However, we must maintain the “prior contact” hypothesis since the Faroe Islands would have been exposed to contact with Europeans since the 4th century, while the islands of Fiji were discovered by European explorers in the 17th century, but it was not until the 19th century that Europeans settled there (English colony in 1874). More than colonizing or culturing, the arrival of Europeans to America seems to be an “assimilation/annihilation” process, not intentionally but due to the microorganisms that traveled as stowaways with the Europeans. However, the attitudes of the Europeans were also not inclined to avoid such disasters, and they even took advantage of them. An entire continent and its great civilizations were mortally wounded in another enormous change in history that was caused by microorganisms and external travelers.

### 2.4. The 1918 Flu Pandemic: “The Spanish Flu”

The most intense outbreak of infectious disease in human history may have been the 1918–1919 influenza pandemic, known as the “Spanish flu”. This curious name is due to the fact that in this country, which remained neutral during the First World War (1914–1918), news of the pandemic was reported first, while in the warring countries, it was not reported to avoid demoralizing citizens and combatants. The news from Spain about the course of the pandemic reached world public opinion, which began to know of it as “something that happened in Spain”, coining the name Spanish flu, with which this pandemic has gone down in history. This pandemic is only comparable to the Plague of the 14th century, but it developed over a much shorter period of time. It is estimated that around 500 million people were infected (a third of the world’s population at that time) [72]. The pandemic overlapped with the final phase of the First World War and the postwar period, and it produced many more casualties than the war itself: about 50 million, although some estimates speak of 100 million, while the war itself caused about 9 million casualties [24,26]: Most of the deaths from the pandemic occurred within a few months during the fall of 1918. It killed more people in twenty-four months than AIDS has killed in twenty-four years and killed more people in one year than the Black Death killed in a century. However, this was not the Middle Ages, and 1918 marked the first collision of science and epidemic disease. The disease was exceptionally severe, with fatality rates above 2.5%, compared to less than 0.1% in other influenza pandemics [24,73]. In 1918, the cause of human influenza and its links to avian and swine influenza were unknown. Despite the clinical and epidemiological similarities to the influenza pandemics of 1889, 1847, and even earlier, it was surprising that such a fatal disease was actually influenza. In fact, this question was only answered in the 1930s, when closely related influenza viruses (now known as H1N1 viruses) were isolated, first from pigs and shortly afterwards from humans. Seroepidemiological studies soon linked both viruses to the 1918 pandemic [74,75]. We now know that the pandemic was caused by an influenza A virus of the H1N1 subtype, named for the glycoproteins hemagglutinin (HA) and neuraminidase (NA); to date, 18 different HA and 11 NA subtypes have been identified [76]. The influenza virus genome is made up of eight RNA molecules that encode HA, NA, and the other virus proteins. In infections that are caused by more than one virion subtype, these molecules can be rearranged into new subtypes [77]. The sequencing of the genome of the 1918 H1N1 pandemic influenza virus was not completed until 2005, which was achieved by Taubenberger et al. [78] after a 10-year effort. The HA1 domain of the HA gene was sequenced [79] from three victims from the 1918 pandemic who had been buried in a permafrost grave in Brevig Mission, Alaska (United States of America); one of these victims was an obese female whose lungs were perfectly frozen and preserved in the Alaskan permafrost. In the following years, the sequences of other segments of the 1918 virus genome were published, until its entire coding sequence was completed in 2005 [25,80,81,82,83].

The impact of this enormous pandemic was not limited to 1918–1919. All influenza pandemics since then, and indeed nearly all influenza A cases worldwide (except human infections with avian viruses such as H5N1 and H7N7), have been caused by descendants of the 1918 virus, including viruses that are H1N1 “derivatives” and the H2N2 and H3N2 viruses. The latter are composed of key genes from the 1918 virus that have been updated by later proteins, making the 1918 virus of the “mother” of all 20th century flu pandemics [24]. For nearly a century, the question has been raised as to when, where, and how the eight genomic segments encoding HA, NA, and the other virus proteins entered the human population to cause such brutal devastation and in such a short time, with a particularly severe impact being seen among young adults in their 30s [84]. This severity in young people today is explained by the contacts of the world population of 1918 with different subtypes of the virus in previous pandemics, such as the so-called “Russian flu of 1890”, which was proposed to have been caused by the H2N2 subtype [85]. This pandemic was first reported in Saint Petersburg (Russia) in December 1889 and spread rapidly around the world. Although it started as a relatively mild disease in Europe and North America, death tolls increased by late December 1889–January 1890. Less data are available about Asia and the Southern Hemisphere, but it is estimated that the “Russian flu” caused about 300,000 deaths worldwide [86]. The Spanish flu presented two very different phases during 1918: the first phase began in the spring and lasted until August and comprised short-term illness and relatively few deaths, but starting in the fall, a much more severe second phase occurred [24].

It is still not clear when and where this pandemic may have started, though at least three scenarios have been proposed. The American doctor in a rural area of Kansas (USA), Loring Miner, reported the first cases in the first weeks of 1918. They were 18 cases with flu-like symptoms, but with higher mortality (three deaths). This area was located a few hundred miles from a US Army camp, where one of the first recorded outbreaks of the pandemic was reported only a few weeks later [34]. However, it seems that everything had started earlier. In December 1917, 14 of the 16 existing military camps were affected by influenza, with almost 250 deaths being recorded between October and November of that year [34]. As an example, this fragment of the text by Callender and Copal from 1929 [87] states that “*Camp Dodge was a National Army cantonment and received its first increment of troops between September 1 and 15, 1917. Promptly upon opening the camp, measles, together with other acute respiratory diseases, made its appearance in epidemic proportions, as is indicated by Chart VII. The case fatality rate increased from 0.64 per cent in July, to 3.85 per cent in August, preceding the occurrence of the pandemic of influenza. Definite influenza cases appeared in considerable numbers in September, when the case fatality rate increased to 5.40 per cent and reached its peak for the war at this camp in October, when it was 6.28 per cent”*. Obviously, this information did not reach the general population, and it was Dr. Miner who first gave the evidence of the epidemic outside of the military environment. In order not to damage the course of the war, advised by his military commanders and despite the news of sick and dead soldiers on the ships when the flu was declared on board, President Woodrow Wilson did not suspend the shipments of troops to Europe, probably contributing to the spread of the epidemic [34]. Wilson himself contracted the disease in the armistice negotiations in 1919 in Paris. However, against the possibility of the American military introducing the flu into Europe is the fact that the first documented outbreaks occurred in a British army camp in Etaples, France, in late 1916 and early 1917, while the United States entered the war in April 1917. The Etaples cases were documented by Hammond [88], who observed an outbreak of “purulent bronchitis”, which was confirmed by an outbreak in southern England shortly thereafter.

The possibility that this pandemic originated in China has also been considered, once again considering the hypothesis of “coming from the East”, which has often been considered in many of the historical pandemics that we have already discussed. According to this hypothesis, the virus would have been brought to Europe by tens of thousands of Chinese workers who had been recruited by the British and French to work behind the lines in France during the First World War. On their way east to France, these Chinese workers passed through North America, arriving in Canada. Could they be the ones who carried the virus to North America and then to Europe? [89]. Mortality in the Chinese city of Canton (Guangzhou) during the 1918–1919 influenza outbreak was relatively low, suggesting previous exposure to the virus responsible for it or to a relative that was close enough to it, which may have been in circulation in southern China for some time before 1918 [89]. Once they arrived in Europe, these Chinese workers were in the Montreuil area of France during the 1914–1918 war, just ten kilometers away from the Etaples camp, which was where the severe influenza outbreak among the British troops was reported in 1916–1917 [88]. Following the hypothesis of Chinese origin, previously, in 1888, an influenza epidemic had been reported in Hong Kong [89] that may have been the origin of the “Russian flu” mentioned above. Influenza type A viruses usually have their primary hosts in animal reservoirs (especially avian), and pandemics are characterized by the interspecies transmission of new variants to humans [90]. In this sense, in favor of the hypothesis of the Chinese origin of the pandemic, it has been argued that in China, agricultural practices put ducks, pigs, and people in relatively close contact, which suggests a zoonotic origin, as is also the case with many of the pandemics that have been seen in recent decades (coronavirus SARS, MERS, etc.) [91]. This hypothesis gains strength if we consider that the 1918 pandemic viruses were the hybrids with seven of the eight segments with a low Uracil content, something that is characteristic of viruses of avian origin [92]. It must be remembered that avian influenza viruses are not transmitted directly to humans, which is probably due to the characteristics of the sialic acid receptors on the surface of the cell that bind the HA protein, as reviewed by Taubenberger and Morens [24]: Bird-adapted strains of the influenza virus preferentially bind to sialic acid receptors with α-type (2–3) galactose-bound sugars, whereas human-adapted influenza viruses preferentially bind to α (2–6)-linked receptors [93,94,95]. Changing this avian receptor configuration requires the substitution of a single amino acid, a substitution exhibited by the HAs of sequenced viruses from the 1918 pandemic, suggesting that this was a critical step in the adaptation to the human host [23]. Additionally, some of the 1918 flu viruses had undergone a second change that increased their ability to bind to human receptors [96]. These different preferences for sialic acid receptors represent a barrier to switch from bird to human hosts. On the other hand, the epithelial cells of the trachea in pigs contain both types of sialic acid linkage (α (2–3), α (2–6)), so they are susceptible to both avian and human influenza viruses and may support their rearrangement and evolution: In this way, when a pig is simultaneously infected with a virus of avian origin and one of human origin, the resulting genomic segments can be rearranged when they replicate, giving rise to “hybrid” viruses, with new subtypes appearing that infect humans [97], which would be the most plausible hypothesis for the origin of the H1N1 subtype that was responsible for the great pandemic of 1918 [92]. Based on these data, it seems plausible that a pig was infected by influenza viruses from a duck and a human on a Chinese farm, causing the rearrangement of the genetic fragments and the birth of the H1N1 pandemic. However, in the case of the H1N1 influenza pandemic, Chinese workers were also sent to Europe from the east, through the Suez Canal, routes that were abandoned as early as March 1917, when they were transported through Canada on sealed trains, so it would be unlikely that they cause infections in Canada during these trips and that they were thus the original hosts of the virus [34].

**Figure 4:**
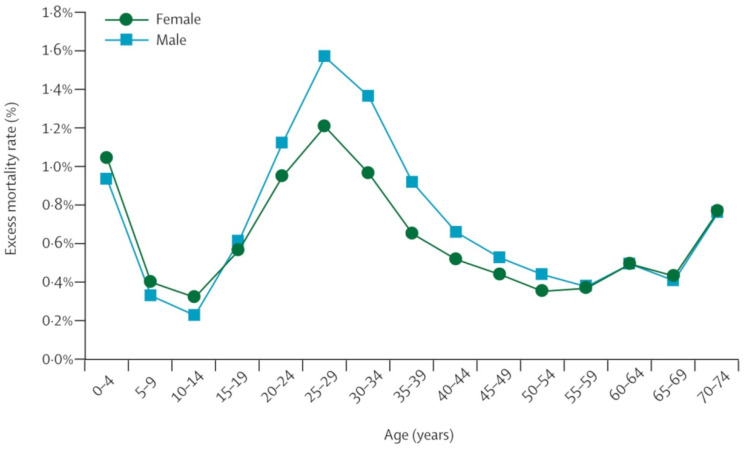
Median excess mortality rate by age and sex for the 1918–1920 flu pandemic based on data from 13 countries with available data. Source: This figure was published in *The Lancet*, 368, Christopher J L Murray, Alan D Lopez, Brian Chin, Dennis Feehan, Kenneth H Hill, Estimation of potential global pandemic influenza mortality on the basis of vital registry data from the 1918–20 pandemic: a quantitative analysis, 2211–2218, Copyright Elsevier Ltd. 2006 [84].

Before the Etaples cases, in 1916, a German newspaper reported cases of *“pure flu fever, without catarrhal symptoms and with general symptoms, such as headache and loss of appetite”* [34], but it cannot be ruled out that these cases corresponded to the so-called “trench fever” caused by *Bartonella quintana* that was detected in the bodies of Napoleonic soldiers after their retreat from Russia [36]. Of all of these possibilities, the best documented case is the outbreak of purulent bronchitis in Etaples [88] in late 1916 and early 1917. This study described a *“symptom complex so distinctive that it constitutes a definite clinical entity”*, providing what may be the first clear account of the pandemic disease that peaked in 1918 and became what is known as the Spanish flu. With great clarity, the authors described the clinical features and rapid progression of the disease, which typically resulted in death from asphyxia caused by pus-filled airways [88]. As previously indicated, due to the dates of these cases, the American origin can be doubted since the United States entered the war in April 1917. This tremendous pandemic especially affected young individuals, a phenomenon that had already been observed in early 1918 in New York [98], producing a curious W-shaped mortality curve, with the mortality peak among middle-aged people, instead of the expected U-shaped curve, with deaths mainly occurring among the very young and the elderly (Figure 4) [84], which will be commented upon later. These data suggest the very early presence of the pandemic in the US. How did it get there so early if it originated in Etaples, France? Was it the shipments of troops from the United States, who entered the war on April 6, 1917? Even if there were early cases in France and the United Kingdom in 1916 and 1917, this still would not necessarily indicate that the pandemic virus first emerged in Western Europe. Phylogenetic studies indicate that most of the avian genomic segments in the 1918 human H1N1 virus appear to be from the Western hemisphere and that they are probably North American in origin [92] and that the HA gene of the H1N1 pandemic virus had already been circulating in the human population since long before 1918 [99], returning the ball of the origin of the pandemic to the court of American soil. However, if this was the case, who transported it to Europe so early if the US officially entered the war in April 1917? This question remains unanswered.

The rearrangement of the gene segments of the virus that gave rise to the pandemic probably occurred in around 1915, according to the dating of the common ancestor of the human and porcine H1N1 genomic segments and even of the human, porcine, and avian segments [99], and thus long before the Kansas cases or those of the multiple military camps of 1917 [34]. It should be noted that the Northern Hemisphere influenza of 1915–1916 was particularly intense in the US [100], but we cannot know whether these viruses were of the pandemic H1N1 type, as we do not have any phylogenetic evidence regarding this possible pandemic H1N1 identity. Additionally, if they were, how did they get to Europe? Since the beginning of 1918, before the pandemic was recognized, viruses with genomic sequences that were identical or almost identical to those of the declining wave of the 1889 “Russian flu” pandemic were already circulating [101]. This pandemic caused considerable mortality in multiple waves, for instance, in London, it displayed three great waves from 1889 to 1892 [86]. The question remains as to how these more or less isolated but not massive outbreaks became the tremendous pandemic that hit the entire planet? In this regard, the planetary spread of the 1918 pandemic was able to occur in a similar way to the spread of other pandemics throughout history: the movement of large population masses seems to be the most likely cause. The demobilization of the soldiers who participated in World War I precipitated massive outbreaks around the world. More than two million men and women from the United Kingdom, Canada, Australia, New Zealand, China, India, the West Indies, South Africa, Fiji, Portugal, and elsewhere, who served in the British Army in northern France, returned to their countries, possibly causing the rapid worldwide spread of the deadly virus and the beginning of its most intense wave. Moreover, hundreds of thousands of workers from neutral countries, who worked in the warring countries, were also repatriated, bringing the virus to their countries of origin. This was the cause of the pandemic in Spain [34].

As commented upon before, one of the most relevant clinical data points of this pandemic is that it affected young adults very severely and how this connects the experience of 1918 with previous pandemics. Fundamentally, the so-called antigenic imprint, a kind of “original antigenic sin” caused by early childhood exposures to one or the other of the two HA phylogenetic clusters, seems to explain this curious spike in mortality among young adults in 1918 as well as the minimal mortality seen in those who were a little younger or a little older: the curious “W” shape of the mortality curve by age resulted from this pandemic compared to the classic “U” (Figure 4) [84,99]. In fact, the year of birth reflects the first strain of influenza A virus that one can be exposed to in childhood, and the very high mortality of young adults in 1918 (the peak of the W) could be explained by the exposure of that same cohort to a putative pandemic H3N8 virus (Group 2 HA) that emerged in 1889 and that would have been replaced by an H1 strain in around 1900 [99,102]. In this way, individuals who were 28 years old in 1918 would have been exposed to a group 2 HA (that is, H3) in childhood that did not coincide with the group 1 HA (H1) of the pandemic virus of 1918. In contrast, most people who are slightly older or a little younger would have been exposed to a group 1 HA as children and would later benefit from relatively good immunity to this and to the pandemic virus, which was an H1N1 virus (with a group 1 HA) [99]. Although the young adult mortality rate from the 1918 virus peaks sharply in those born very close to 1889, it included those born up to a decade before 1889 and up a decade after, which is possibly because a new H1 virus appeared in the early years of the 20th century, displacing the H3 virus of 1889 [99].

#### Consequences of the 1918 Flu Pandemic

The world has changed profoundly in the century since the great pandemic of 1918. This is logical given the long period of time, especially as the rate at which changes occur within society seem to be increasing, with some of these changes having been due to the 1918 pandemic. A century ago, medicine and science were much more limited in their ability to treat disease. Treatments for infectious diseases were limited, but, above all, there were no public health systems, even in developed countries. Health was a luxury since doctors were independent or funded by charitable or religious institutions. Health was a largely inaccessible good, especially in the poorest countries, where the pandemic was most implacable: 17 million people died in India, many of whom were Hindus (7 times more than British residents: 6.2% vs. 0.9%), while in the US, 550,000 people died [34,84]. In fact, the death rates in India were vastly higher than they were in any other country in the world [84], something that seems to be repeating in the current COVID-19 pandemic. The extension of health care and its prioritization are probably the most beneficial effects of that disastrous pandemic, which when combined with the war, probably sowed the seeds of the welfare state in many parts of the world. As has been indicated during other historical pandemics, they seem to show a society that is becoming more just and equitable. Below, we discuss a few consequences for the history of humanity that derived from that pandemic, though not all of them are.

Probably the most positive consequence of this tremendous pandemic was the consolidation of the concept of “health for all”, or at least for a significant part of the population. Unfortunately, a century later, a very large proportion of the world’s population still does not have the benefits of something that is so essential. However, returning to the historical scenario, which is perfectly described by Laura Spinney [34], health authorities learned from the pandemic that it no longer made sense to blame an individual for contracting an infectious disease or to treat it in isolation. In the 1920s, many governments adopted the concept of socialized medicine: free health care for all. Britain and Russia established health insurance systems in the first decade of the 20th century, and many western and central European countries would do so in the following decade as a direct consequence of the 1918 pandemic. Russia was the first country to implement a centralized and fully public health care system in 1920, especially for the prevention of epidemics and famines. After his 1917 revolution, Lenin himself promoted its creation and was aware of the price that had been paid for the triumph of the revolution: famines, epidemics, and civil war had almost annihilated the working class. In 1948, Great Britain created the National Health Service (NHS) which pioneered geriatric care. Some media considered this measure a synonym of socialism, which is why the US probably does not yet have a universal health care system. Ministries of Health were created in the years immediately after the pandemic, providing proof of the inclusion of health in the general policy of many countries and a direct consequence of the great pandemic of 1918. However, in Nazi Germany “public health” became a double-edged sword, with the passing of the so-called Sterilization Act of 1933 [103].

Epidemiology was born as a scientific discipline in the years following the pandemic, as the notification of health data became more systematic [104]. In 1930, the National Quarantine Service was created in China, which oversaw quarantine measures in all major ports in China [34]. Persia multiplied the budgetary allocations for the country’s sanitary infrastructure twenty-five fold between 1923 and 1936 [105].

In general, the state of the health of a nation was considered as an indicator of its modernity and degree of civilization. However, this did not reach the European colonies so much as it reached the citizens of the colonizing powers themselves. The news of the health policy of Russia and the clear marginalization of “colonizers” to “colonized” caused the logical discontent of the colonized indigenous populations and would become the germ of the conflicts that would end up exploding colonial policies, resulting in many new nations being born [34]. The International Office of Public Hygiene founded in Paris in 1907, which merely had an informative role, would give way in 1919 to an international office based in Geneva with an express role of fighting pandemics. In the early 1920s, the League of Nations itself would create its health organization, which would be the precursor to the World Health Organization, which would emerge after the founding of the UN [34].

Leaving public health aside, the 1918 pandemic also contributed to the popular classes and the colonies becoming aware and making themselves heard. Although the Russian revolution of 1917 was a trigger for the labor movement, which has profoundly marked contemporary history, the flu that took hold among the poorest classes fanned the flames by exacerbating a lack of supply that was already very serious and highlighting the terrible class inequalities, which produced a wave of worker strikes and anti-imperialist protests around the world in the fall of 1918. Even in Germany itself, there was a revolution in November 1918, and Switzerland was on the verge of a civil war [34]. This same situation awakened anti-colonial movements, initiating a very important historical change at the sociopolitical level, especially for the British Empire; despite being the winner of the war, the flu was more decisive than the war itself for British colonial policy. In March 1919, an uprising began in Egypt against their English colonial masters when the Egyptians became aware that their death rate was double that of their British “protectors”. This uprising culminated with the independence of Egypt in 1922 [34]. In India, these tensions were aggravated due to the flu (17 million people died, most of whom were Hindus, with a mortality rate 7 times higher than that of British residents). Nationalist movements, including Mahatma Gandhi himself, who was also infected, blamed the British rulers for this disaster. During the worst phase of the pandemic, there was a great drought, yet the entire British government maintained the export of wheat until October, where at the height of the pandemic, the flu was joined by cholera [34]. Furthermore, even after the war, the martial law that suspended civil rights was extended, unleashing a wave of riots, with hundreds of deaths resulting from British repression [34]: war, flu, and repression: an indigestible menu that produced the definitive estrangement of the Hindus from their British protectors until Rabindranath Tagore himself, a recipient of the Nobel Prize in Literature, renounced his knighthood in protest to the massacres in the city of Amritsar. However, there was a long way to go until India achieved independence, which they would achieve in 1947.

Moreover, although the epidemic did not cause radical changes in the social structure (not as great as the fall of feudalism due to the Black Death in the 14th century, for example), it was fundamental to moving the social model towards a more balanced position between genders in many countries. The enormous death toll of the war and the pandemic had a disastrous economic impact. In addition, both disasters preyed on young men, to the point that in many countries, there were no young men left to run the family business, run farms, or train for professions and trades, making it difficult for millions of women to find a partner to marry and have children with to replace the millions who had died and to follow the traditional social model of the man as the breadwinner and the woman as the organizer of the family home. In this way, the lack of workers that was caused by the flu and the war gave women access to the labor market, giving them better wages. In 1920, women accounted for 21% of all of the employees in the United States, and in the same year, the nineteenth amendment to its Constitution ratified the right to vote for women [106].

As indicated, the Black Death had long-term economic, social, and cultural consequences, which shaped social behavior well into the 20th century. Similar to the Black Death, the Spanish flu also had long-lasting social consequences, leading to a decline in social trust. This would result from having experienced the social disruption and widespread mistrust that characterized the pandemic period. Aassve et al. [107] have recently evaluated the effect of the Spanish flu pandemic on social trust and its almost permanent effects on society among the descendants of the survivors of that catastrophe. The results of that evaluation suggest that the least amount social trust was passed on to the descendants of Spanish flu survivors who emigrated to the United States. Since trust is a crucial factor for long-term economic development, it represents a long-term negative effect of that pandemic and warns us of what may happen after current and future pandemics.

**Figure 5:**
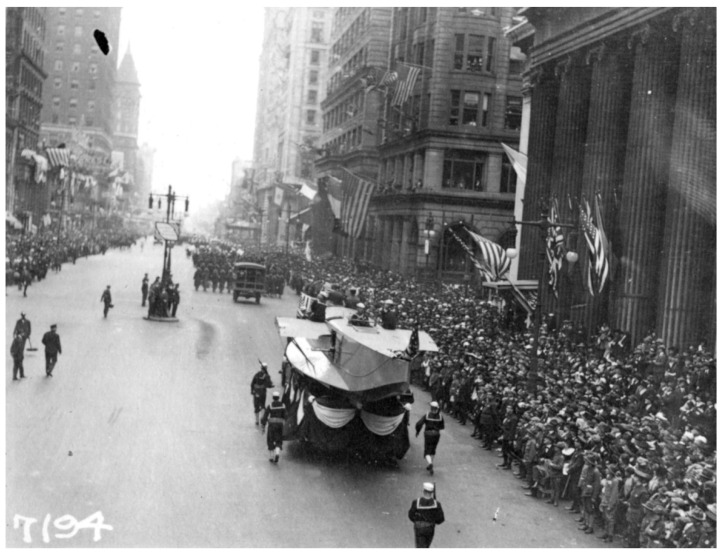
A naval aircraft factory float, with the hull of an F5L patrol seaplane, moves down South Broad Street during a parade in Philadelphia on 28 September 1918. Source: NH 41730 courtesy of the Naval History and Heritage Command [108].

At a purely health-related level, after the pandemic, a sense of discontent with medicine and with science as a whole became general [34]. This led to a considerable boost for “alternative medicines” such as homeopathy, which was included in the pharmacopoeias that were even adopted by conventional doctors. Sunlight and clean air became synonymous with health, and by 1930, the concepts of nature and cleanliness were firmly linked. In contrast, the anti-smoking movement collapsed after the war. Smoking became fashionable, as tobacco was considered to have beneficial properties. In this environment of distrust of scientific medicine, religious movements also emerged that claimed that prayer alone had proven superior to conventional methods. In October 1918, the American city of Philadelphia suffered especially badly from the flu, and the newspapers proclaimed the failure of science [108], resulting in religious moves such as the Philadelphia-based Tabernacle of Faith proposing *“Witnesses to God’s divine healing*”. However, an interesting episode related to this city should be remembered here. In September 1918, several US towns organized parades to promote war bonds, the sales of which would help finance the conflict that had not yet fully ended. Two of these cities, Philadelphia and St. Louis, took diametrically different measures regarding the parade once the first cases of the disease were known: St. Louis chose to cancel it, but Philadelphia decided to go ahead with the event. A month later, more than 10,000 people had died from the flu in Philadelphia, and just 700 had died in St. Louis [34]. It seems obvious that maintaining social contact in Philadelphia was the cause of the enormous death toll, but the “Tabernacle of Faith” considered that prayer saved those who did not die. This non-objective way of interpreting reality is reminiscent of earlier times and pandemics, such as the Plague of the 14th century (Figure 5).

In fact, an analysis of the interventions in various US cities during 1918 showed that those municipalities that banned mass gatherings and that closed theaters, schools and churches had fewer deaths, making the benefits of confinement clear. Any scientist who was witness to the pandemic understood that good science required an open mind, experimental rigor, and a healthy dose of humility and would remember the reflections of Émile Roux (co-founder of the Pasteur Institute) on the *“organisms whose existence can only be deduced from their effects”* [34], a perfect allusion to microorganisms. Virology was established as a discipline, the first flu vaccines began to be produced, although they were not available until the 1940s, and Fleming discovered penicillin while trying unsuccessfully to culture Pfeiffer bacillus (*Haemophilus influenzae*), which doctors from a century ago thought was responsible for the influenza epidemic [34].

### 2.5. Yet Another Tremendous Pandemic Disease: COVID-19, Coronavirus SARS-CoV-2, a “New Kid on the Block”

We entered the 21st century with significant advances in the field of genetics by having completed the sequencing of the human genome, new generation sequencing platforms, fast automatized serological and antigenic tests, great advances in new strategies for vaccine creation, and the development of highly effective antiviral treatments. Of note are the simple and tolerable combinations of direct acting antivirals against the hepatitis C virus (HCV) that have revolutionized the field and that have opened up the possibility, for the first time in the history of medicine, that in the span of a lifetime, we might be privileged spectators of and modest actors in the discovery in 1989 [109] and in the elimination of the virus as a public threat by 2030 [110]. In this scenario, virology experts studying the extremely high capability of these viruses to generate variation and to thus avoid treatments and vaccines have suggested that the possibility of a pandemic caused by an RNA virus should be taken into serious consideration in diverse forums [111,112]. Even the magnate Bill Gates had already stated in 2014 that we were not ready for the next pandemic [113], as was shown by the end of 2019, in which even the most pessimistic could not have foreseen the magnitude of the impending tragedy. This new pandemic was caused by a new virus: a coronavirus named SARS-CoV-2. This name was selected because of its similarity to SARS-CoV, which was first described in November 2002 in Guangdong province, China, where it infected 8096 individuals, 774 (9.6%) of whom died [114]). Today, the SARS-CoV-2 virus pandemic is still on the rise, with hundreds of millions infected and millions dead (data continuously updated by Johns Hopkins University [115]). According to the estimations of the Institute for Health Metrics and Evaluation, University of Washington, the numbers are probably higher, [15]. However, the current rate of vaccination may cause all predictions to be thrown out.

The first outbreak of the novel SARS-CoV-2 coronavirus was reported on 31 December 2019 by the Wuhan Municipal Health Commission of China and was said to have originated in the Huanan Seafood Wholesale Market of Wuhan, Hubei Province. According to Chinese government data, the first person to show signs of the new infection was a Hubei resident aged 55, who was reportedly infected on November 17. The first case was diagnosed at Wuhan Central Hospital on 16 December 2019. However, “patient zero” has not yet been identified. From that date onwards, new cases of the still unreported disease rose day by day in China until December 27, when Dr. Li Wenliang, Dr. Ai Fen, and Dr. Zhang Jixian alerted the public and reported to China’s health authorities that the disease was caused by a new coronavirus. This new disease was labelled as COVID-19, representing the COrona VIrus Disease, from the year 2019. Although the outbreak was first identified in the Huanan market, many early cases were found in other markets, and some were found outside of any market, meaning that the role of Huanan market as the origin of the outbreak cannot be confirmed [21].

Extensive next-generation sequencing (NGS) experience was crucial for the successful sequencing of the entire genome of this new SARS-CoV-2 virus on 12 January 2020, and the genetic sequence was publicly shared and then uploaded to the NCBI databank on 17 January 2020, codified as MN908947.3 (isolate Wuhan-Hu-1). Very early on, angiotensin converting enzyme 2 was identified as the main cell receptor. In fact, this is the same receptor that was previously described as being used by SARS-CoV [116]. Since 13 January 2020, when the first case of COVID-19 in Thailand became the first recorded case outside China, the virus has spread rapidly through the human population, with a very high sequence identity (99.9%) between the isolates that have been recovered from all over the world [117].

#### 2.5.1. New Opportunities Are Born from All Crises

All around the world, groups with extensive experience with NGS started a race to sequence the virus genomes that had been isolated from their infected citizens using all of the available resources, as NGS is the most accurate tool for genomic viral studies [118,119]. Viruses cannot be walled-off, which is a great challenge for public health; we can only build high-throughput technological walls based on knowledge. In this regard, the genetic detection, identification, and characterization of infectious agents are the first steps to confronting their continuous challenge. In the heat of the first pandemic wave, all of the researchers who had something to contribute to the knowledge and to finding a solution to the terrible pandemic started working in collaboration, sharing results on open-source data-sharing platforms and open pre-print platforms, of which medRxiv and bioRxiv are the most popular. The whole scientific community can be considered to be acting as a “single research team” within a global network promoting a planetary exchange of scientific knowledge, which is probably one of the most relevant and positive side effects of this terrible pandemic and is one that will help us to face new pandemics and to reduce their terrible consequences. This huge scientific effort has resulted in more than 120,000 articles only one year after the start of pandemic, with this number based on a simple search in PubMed using the search term “COVID-19 pandemic”.

The open-source publication of the SARS-CoV-2 genetic composition allowed for the rapid design of multiple molecular diagnostics technologies [19], especially high throughput Nucleic Acid amplification tests (NAT) and diagnostic tools based on RT qPCR such as the completely automated Cobas 6800/8800 (Roche) system or Transcription Mediated Amplification (Hologic/Grifols), which have contributed to performing millions of tests in just months [120,121,122], significantly increasing the molecular diagnosis capacity of thousands of diagnosis centers. In our case, the daily NAT tests have increased 25-fold compared to 2019, with an average of 5000 daily NAT tests being conducted in 2020. Indeed, the term “PCR”, was previously restricted to scientific language, but it has since entered the common language. It is important to highlight that this improvement in molecular testing capacities should be maintained to implement effective control measures for limiting the spread of infection, preventing vaccine resistance, controlling SARS-CoV-2 variants, and for future pandemics. Moreover, many laboratories have increased their sequencing capabilities.

The sequence repository Global Initiative on Sharing All Influenza Data (GISAID) data-sharing platform [123] has been crucial for uploading and sharing sequences from most nations and territories worldwide. GISAID and Nextstrain [124] are high quality tools that can be used for sequence display and analysis have been critical to studying origins, epidemiological studies, and tracking viral variant transmission routes [125]. To date (September 2021), more than 31 million genomes have been uploaded to the GISAID [126,127].

The increase in the power of massive sequencing using the ARTIC tool [128] has been crucial for the partial and whole-genome sequencing of SARS-CoV-2, allowing for the identification of variants and relevant mutations with different transmission and pathological capabilities, an essential task for the surveillance of this pandemic [129,130]. Among these variants, special attention should be paid to those associated with extensive transmission, known as “variants of concern” (VOC), the most important of which are currently the variants that were first seen in the UK (Alpha, B.1.1.7); in South Africa (Beta, B.1.351); in Brazil (Gamma, P1); and in India (Delta, B.1.617.2) [125,130,131,132]. NGS has become an essential tool for epidemiological surveillance; for the development of vaccines, antivirals, and novel diagnostic tools; and to monitor the mutations that have arisen in the population that may compromise their effectiveness or that may impact the transmissibility and pathogenicity of the virus and viral dynamics in a chronically (large) infected patient [18].

The synergy that has been created between the high throughput sequencing centers for the early detection, identification, and characterization of new VOC and the diagnostic companies providing real-time RT-PCR-based solutions for the rapid detection of new variants is a nice confluence of interests for improving the control of pandemics. However, probably the greatest human achievement in this pandemic has been the development of highly effective COVID-19 vaccines in record time, especially those that are based on the revolutionary mRNA technology developed by Dr. Kariko [133,134,135]. In fact, the first of these vaccines took just 69 days after the identification of SARS-CoV-2 to reach the clinical-trial stage [136]. This is an extraordinary achievement considering that it typically takes decades to develop, test, and bring a vaccine to market; for instance, this process took 34 years (1954–1988) for chickenpox, 15 years for human papilloma virus (1991–2006), 9 years for measles (1954–1963), 7 years for the first polio vaccine (1948–1955), and 4 years for mumps (1963–1967) [137]. Fortunately, in contrast, mRNA and DNA adenovirus-based vaccines for COVID-19 have achieved agency (FDA and EMA) approval for clinical use in less than a year, with efficacies of 95% for Pfizer-BioNTech, 94.1% for Moderna, 76% for Oxford, 72% for Johnson and Johnson, and 96.4% for Novavax [17]; by 18 May 2021, 1.5 billion people had been vaccinated all over the world, a number that was obtained according to a French press agency count [138].

The Internet has become an incredibly potent tool for sharing new scientific achievements and for facilitating online communication between people, thus preventing physical contact, which is required for viral transmission. Videoconferences replaced close human-to-human contact in familiar, business, school, and many other human activities by means of different online platforms (Zoom, Teams, Google Meet, Livestorm, etc.). Moreover, the Internet and social media have been used as a tool for predicting periodic flu epidemics [139], and this has recently been applied to the prediction of COVID-19 pandemic waves through the use of different social media applications such as Twitter and Google Search [140].

#### 2.5.2. What about the Virus?

The SARS-CoV-2 viral genome is close to 30 kb in length [20], one of the largest genomes for an RNA virus, and is similar to other coronaviruses [141]. This genome requires a balance between stability and diversity, which is important for non-structural protein (nsp) 14, which plays a role in RNA proofreading and repair functions because of its 3′-5′ exonuclease activity, which can correct any mutations that have been introduced by its own RNA-dependent RNA polymerase, which is called nsp12. Nsp14 might have an impact on the high fidelity of replication [142,143,144], thus giving SARS-CoV-2 lower mutation rates than other RNA viruses [145,146]. However, although SARS-CoV-2 is less diverse than other RNA viruses, such as HCV, HIV, and even a DNA viruses such as HBV, its low mutation rate is compensated for by a large number of infected individuals and a high rate of viral production, in which each infected person carries a total of 10^9^ to 10^11^ virions during the peak of infection [147]. In the absence of pre-existing human immunity, the virus has been able to rapidly spread through the human population with low selectivity, showing very little genetic variability between the isolates that have been recovered from all over the world [117]. Currently, the nucleotide homology between the original Wuhan-Hu-1 sequence and a Delta mutant (B.1.617.2) is close to 99.9% (based on our own experimental data). With the large number of actively infected individuals and the global spread of the virus, new variants are continuously appearing as a natural source of variation that is generated during viral replication. The question is whether these new vaccines will protect against the novel variants since vaccines have a fixed composition. Currently approved vaccines do not prevent viral infection, but the virus-specific memory B and T cells that are generated after vaccination [148] protect the vaccinated individual from severe infection and death. Moreover, hundreds of new vaccines are being developed and/or are under development [149], and this will increase the chances of successfully facing the new challenges that new variants will produce.

#### 2.5.3. What Is the Origin of SARS-CoV-2?

Controversy surrounding the origin of SARS-CoV-2 is still in the spotlight. For the serious experts in the field, there is no doubt about the zoonotic origin of SARS-CoV-2 [20,21,22]. Although coronaviruses have been cultured in laboratories all over the world for decades, the probability that an expert would have imagined and precisely mutagenized a previous clone to create SARS-CoV-2 is almost zero: the distance between the closest bat coronavirus (RaTG13) and SARS-CoV-2 implies more than 900 nucleotides. Moreover, in relation to other closely related coronaviruses, SARS-CoV-2 shows the insertion of four polybasic amino acids (PRRA) at the furin cleavage site, and five amino acids (F484, Q493, S494, N501, and Y505) changed in the receptor binding domain in relation to the closest RaTG13 [20] and in other substitutions compared to bat and pangolin coronaviruses [20,150,151], which is not obvious for any expert in the field.

The probability that the cross-species jump resulting in a human pandemic increases as a function of the number of events, and it is highly improbable that this could happen as a single event. As a result, the SARS-CoV-2 pandemic is probably a result of multiple jump events from animal to human (zoonosis) and from human to animal (zooanthroponosis) [152], in which the virus might have evolved in both species, with the subsequent spread among the human population in the absence of pre-existing human immunity against SARS-CoV-2 [153].

In fact, a zoonotic or enzootic origin of pandemics is not exceptional, according to the data reported throughout this text. In fact, in recent decades, the rate of outbreaks of emerging, re-emerging, or new infectious human diseases has been accelerated by certain factors that reflect the expansion of our global footprint and travel network [154], such as the degradation of natural and wild spaces (deforestation, dam construction, changes in wildlife migration patterns, floods due to changes in river boundaries, global warming, climate change causing the expansion of viral vectors, agriculture), bringing humans closer to wild animals, putative viral reservoirs (wild animal parks, hunting, tourism), globalization effects (human mobility losing the quarantine effect, transport of goods, long distance transport of birds and livestock, urbanization, concentrating millions of people in small places), the mass exploitation of animals who are in close contact (pigs, chicken, birds, etc.) including wild animal farms for fur and food production, and health and social activities (transfusions, organ transplants, health care, social changes related to sex and drug abuse, the use of air conditioning, large concentrations in closed halls, stadiums, pavilions).

In addition, all previous scientific data very strongly support the zoonotic origin of SARS-CoV-2, as also reported for other related CoV such as SARS-CoV and MERS [20,22,150,151,155,156]. Some animals (marketplace-masked palm civet, raccoon, dog, and ferret badger) harbor viruses that are almost identical to the genomes of human SARS-CoV [157,158,159] and were likely the intermediate host animals between the original coronavirus, which has been found to infect bats (*Rhinolophus affinis, Rhinolophus sinicus)* and humans. MERS-CoV (still an active infection since it was first reported in 2012, with 35% mortality; 858 deaths from 27 countries [160]) was isolated from humans; MERS-CoV from Dromedary camels and from humans have a >99% identity [141] and are the intermediate animal host between the original coronavirus infecting the Egyptian tomb bat and humans.

The question remains as to how this could have happened and which animals (wild animals, pets, etc.) may have acted as the intermediate link between bat CoV and human SARS-CoV-2. It has recently been hypothesized that the intermediate host for SARS-CoV-2 is linked to the wild animal farms that are used for fur and food production. These farms have been on the rise in China since the 1990s and provide a livelihood for millions of people; these farms are mainly dedicated to farming the fur of minks, foxes, and raccoons (nearly 60 million animals were slaughtered in 2018) [161]. In fact, this clue came from SARS-CoV-2 outbreaks on mink farms in Denmark and the Netherlands, where the virus was initially introduced by humans, evolved in minks, and spilled back over to humans [162,163,164]. To date, SARS-CoV-2 has also been detected in domestic animals such as dogs and cats, and while there is evidence for efficient transmission between cats, they have not yet been considered to be of major concern [165,166,167], allowing us to rule out pets as the origin of these pandemics. However, we must be aware and avoid mass infection in these animals, which could facilitate viral adaptation with unpredictable results [152,168], as recently reported in the case of a novel canine coronavirus transmission from domestic dogs to humans in Malaysia [169].

The SARS-CoV-2 pandemic has reinvigorated the One Health approach [154], and according to the Centers for Disease Control (CDC) “*One Health recognizes that the health of people is connected to the health of animals and the environment”* [170], meaning that we should integrate humans, animals, plants, and environmental health sectors with multisectoral and transdisciplinary disciplines from biomedical sciences, bioinformatics, veterinary medicine, medics to food and environmental sciences.

#### 2.5.4. What Is Being Changed Directly by the Effect of SARS–CoV-2?

Despite living in the 21st century surrounded by great technological advances that have made it possible to explore Mars in situ with two rovers, *Perseverance* and *Tianwen-1*, which are both moving freely about Mars and have completed several flights with the *Ingenuity* helicopter, before the COVID-19 pandemic, nobody could have predicted that another “invisible enemy”, a simple coronavirus, would be able to stop the whole world.

If we were to highlight three major human pandemics, one would be the 1918 H1N1 influenza pandemic that infected about 500 M people (a third of the human population of 1.8 billion inhabitants), with 50 M deaths worldwide [72]. This pandemic was fueled by the First World War, with the widespread troop movements from America to Europe and the poor health conditions that were caused by the war. The second would be the Black Death in the 14th century, which spread along the Silk Road from China to Europe [10] and during a time when human beings had limited health and technological knowledge. The third would be the 2019 SARS-CoV-2 pandemic, which has occurred during an era with considerable health care know-how and advanced technological power. It is very difficult to establish parallels between those three events and all other previous pandemics, but in all cases, an infectious agent was able to create terrible damage to the human world, and the lesson that we can take away from this is that regardless of the socioeconomic, technological, and political situation, we are at risk of new events, as we are living in a world where geographical frontiers have practically disappeared; mountains, rivers, desserts, and oceans are no longer obstacles for infectious agents, which they were during past pandemics.

It is clear that the tragedy could have been even greater, with 7.8 billion inhabitants in 2020 compared to 1.8 billion in 1918 during the flu pandemic [171,172]. Indeed, we have done something right to mitigate a major disaster. The question is whether any positive lessons can be drawn from this pandemic, and we believe that the answer is “yes”. The first lesson is that the easiest and most effective tool for stopping the free spread of a viral infection is mass confinement (lockdown), which was successfully adopted in the vast majority of countries, generating impressive images of large “ghost” cities and producing a transient decrease in pollution levels [173].

The second lesson is that new available technologies for the rapid detection, identification, and characterization of the infectious agent have been significantly improved, and especially highly effective vaccines have been produced, such as those based on mRNA/DNA platforms, with many more that are currently under development [149]. This has been the result of applying previous knowledge that has been accumulated over years and years of research through important investments from the pharmaceutical industry. At the same time, health care systems have adopted new protocols to take the strain caused by the huge number of infected patients [174,175], discovering the ability to adjust entire hospital departments and facilities in a matter of hours, allowing for a huge increase in the number of ICU beds (in our case at Hospital Vall d’Hebron, for example, we went from an initial 60 ICU beds to more than 200 during the first pandemic wave, with 800 COVID-19 patients hospitalized simultaneously in the hospital’s total of 1200 beds) and in the number if health care teams involving all health care workers. These strategies made it possible to greatly reduce the strain on health care systems in the following pandemic waves, establishing mass preventive screening, organizing vaccinations, and developing accurate quarantine protocols. Many strategies will remain after this pandemic and will be extremely useful for confronting new ones.

The third lesson is the power of human data sharing. All of the researchers with the ability to contribute to finding a solution to this pandemic have made a huge scientific effort (>120,000 articles), with all scientific groups working and sharing results, looking for deeper and fast knowledge, and trying to make the “invisible” enemy “visible”.

The fourth lesson is that despite the outstanding response to this new virus, SARS-CoV-2 has exposed the weaknesses of our system for preventing the spread of emerging infectious agents and their impacts on human health. New opportunities are born from the worst crises, and the world has learnt that the early detection of a novel threat and the implementation of control measures when there are few cases increases the success of such actions to control and manage it. Additionally, as a negative collateral effect, the huge health effort that is required to deal with this situation has required directing most health resources toward this infection, resulting in a detriment to the optimal care of other diseases by means of surgical interventions, doctor visits, treatment prescriptions, preventive controls, etc. The real impact of this must be thoroughly analyzed beyond this terrible crisis and redressing this situation must be a priority. As an example that is close to home, serological studies of viral hepatitis B and C alone saw a 25% decrease in activity between 2019 and 2020 in our laboratory, which serves the whole of the city of Barcelona (1.5 M people). These data suggest a corresponding decrease in the diagnosis of such diseases, considerably hindering the goal of eliminating hepatitis C as a global public health threat before 2024 in Spain [176], as was hoped for before the COVID-19 pandemic. This is just an isolated example, but there are certainly many more.

So far, we have discussed some of the effects of this pandemic on people’s health, but we must not forget that their finances are also being affected. The spread of the SARS-CoV-2 pandemic has strongly affected national economies, especially due to the halting of the economy as a result of lockdown measures to tackle the spread of the virus. Some examples of the economic impact of the COVID-19 pandemic, which were assessed in early 2021 [177], are the big shifts in stock markets, which may affect the value of pensions and individual savings accounts. Due to the dramatic fall of the main financial indexes, central banks in many countries have dropped interest rates to stimulate the economy, making borrowing cheaper and encouraging spending. This difficult economic situation has caused the global economy to suffer “the worst decline since the Great Depression of the 1930s” in 2020, according to International Monetary Fund (IMF) estimates, with China being the only major economy to grow (2.3%). As a result, unemployment rates have increased in major economies; for instance, the IMF estimates that unemployment in the United States reached 8.9% in 2020.

## 3. Microorganisms Are Not Always Enemies: Microbiota, Endogenous Viruses, and as Biotechnological Allies for Millennia

Up until this point, we have reviewed some examples of the consequences of “villainous” microorganisms, relentless genocides that have conditioned our history, causing catastrophe after catastrophe. Despite this, we should also keep in mind that more than 3.5 billion years ago, bacteria were the inventors of all of the chemical systems that essential for life, though at a reduced scale, as stated by Margulis and Sagan in their book *Microcosmos* [178]. This ancient and elevated biotechnology led to the development of fermentation, photosynthesis, the use of oxygen in respiration, and the fixing of atmospheric nitrogen, creating almost perfect ecosystems, such as microbial mats. These microbial communities are structured in horizontal strata that function as a consortium, where biogeochemical cycles and biochemical processes are coupled [179], allowing the waste of one type of microorganism to be the energy source of another. Lynn Margulis once told an interviewer that *“We tend to associate the word bacteria or microbe with disease when they are just life! You are a walking bag of bacteria”*. For example, cows can digest cellulose thanks to the microbial symbionts that are housed in their stomachs, and fish that live on the seabed can host glow-in-the-dark bacteria.

However, if, as Lynn Margulis said, microorganisms are the real entities that are responsible for life on our planet, which means they are practically our “creators”, can we find “friends” among microorganisms? What if we look within ourselves? Some are part of our own genome (endogenous retroviruses) or colonize us as an additional organ (microbiota). Therefore, we can safely say that microorganisms are an essential part of us. Moreover, they have given us the ability to feed ourselves with cheese, bread, wine, yogurt, etc., for many millennia [180].

### 3.1. Microbiota, Our Bacterial Friends

We have commented on the “evil ways” of some bacteria, especially *Yersinia pestis* (Plague), but also *Vibrio cholerae* (cholera) and *Rickettsia prowazekii* (typhus), which have been responsible for so many deaths throughout history, and there are many more. A kind of apocalyptic army that seems to have no other purpose than to annihilate us, a purpose that it has tried to achieve throughout history and that it seems to continuously be trying to fulfil. It is hard to imagine that there are other bacteria that can benefit us in some way, but it turns out that we are colonized by millions of them, and these bacteria allow us to stay alive. We are referring to the bacteria in our intestines, which we call “intestinal flora”; we even use this term in advertisements for nutritional products. We confer the role of great benefactors on these microorganisms, and almost consider “friends”, but we avoid calling them “bacteria”, which is what they are. Perhaps this is to avoid the rejection that the term bacteria has in popular language.

In recent years, the intestinal flora has also come to be referred to as the gut microbiota, a truly living microscopic world that is made up of a bacterial community of around 1000 types of bacteria, with a total weight of 2 kg and that comprises somewhere between 10^13^ and 10^14^ microbial cells, a population that is up to ten times more abundant than our somatic and germ line cells [181]. The genes comprising the microbiota, which is known as the microbiome, harbor between 50-fold and 100-fold more genes than the host does [182]. These numbers are derived from the total bacterial cells in the colon, the organ harboring that harbors the greatest microbe density [183], most of which belong to three major phyla: Firmicutes, Bacteroidetes, and Actinobacteria [184]. On a practical level, it seems that we are more bacteria than we are human. Indeed, the microbiota is considered to be an additional organ in our anatomy that controls the essential functions that are required to keep us alive, such as helping with the regulation of energy and protection against other intruders such as the viruses and bacteria that cause disease [181]. Furthermore, the gut microbiota may also affect the expression of host genes; for example, DNA microarray analysis has shown that the colonization of germ-free (GF) mice with *Bacteroides thetaiotaomicron*, a prominent component of mouse and human gut microbiota, affects the expression of the genes that regulate several pathways such as postnatal maturation, nutrient uptake, and metabolism [185].

The gut microbiota is important for fermenting unabsorbed starch and dietary fiber. Short-chain fatty acids (SCFAs) such as acetate, propionate, and butyrate, are the main fermented end products that are produced in the colon through the bacterial fermentation process of dietary fibers and resistant starches [186]. Microbial-synthesized SCFAs contribute to 70% of ATP production in the colon, acting as one of the energy substrates for the host, thereby contributing extra daily dietary energy that can be used by the host for other metabolic processes [182].

There are a number of potential pathways through which the gut microbiota can influence brain function, namely by means of SCFA, which possess neuroactive properties, exerting crucial physiological effects on the brain. Gut microbiota dysbiosis has been evidenced in behavioral and neurologic disorders, such as depression, Alzheimer’s and Parkinson’s diseases, and autism spectrum disorders [187,188,189,190,191]. In addition, alterations in the gastrointestinal microbiota have also been linked to bipolar disorder. Specifically, bipolar disorder has been linked to decreased Firmicutes, specifically *Faecalibacterium* [192]. The positive modulation of the gut microbiota with the introduction of living microorganisms has been proven to be successful in improving some psychiatric disorders. For instance, Bagga D et al. [193] demonstrated that a formulation containing several probiotic bacteria such as *Lactobacillus casei*, *L. acidophilus*, *L. paracasei*, *B. lactis*, *L. salivarius*, *L. lactis*, *L. plantarum*, and *Bifidobacterium bifidum* altered brain activation patterns in response to emotional memory and emotional decision-making tasks, which were also accompanied by slight changes in the gut microbiome profile. In another study [194] with healthy female volunteers, the intake of fermented milk products with a probiotic (*Bifidobacterium animalis* subspecies *Lactis*, *Streptococcus thermophiles*, *Lactobacillus bulgaricus*, *and Lactococcus lactis* subspecies *Lactis*) for four weeks was associated with the modulation of the responsiveness of an extensive brain network that contains the regions that control the central processing of emotion and sensation. Moreover, communication exists between the immune system, the gut, and the central nervous system, as reviewed by Dinan and Cryan [195]. In addition to producing molecules with neuroactive properties, such as SCFA, certain bacteria within the human gut are involved in immune development, e.g., the microbiota seems to play an important role in the maturation, morphology, and immunological function of microglia in the central nervous system [196]. The manipulation of the microbiota is currently being proposed as a treatment for an ever-widening range of diseases, including psychiatric disorders, through the use of procedures such as fecal microbiota transplantation [197].

Gut microbiota-synthesized micronutrients such as vitamin K2 [198] remain essential in decreasing vascular calcification, elevating high-density lipoprotein, and lowering cholesterol levels, contributing to a reduced risk of cardiovascular disorders [199]. In addition, the gut microbiota also serves as an important source of vitamins B for the host [200]; vitamins B5 and B12 are exclusively synthesized by the intestinal microbiota. These vitamins act as coenzymes for a broad range of host biochemical processes, including in the production of acetylcholine and cortisol, both of which are required for normal nervous system function. A deficiency in these metabolites is linked to several disorders, such as gastrointestinal discomfort, insomnia, and neuropsychological and hematological disorders [201,202]. Moreover, the gut microbiota also plays an important role in the metabolism of bile acids. The 5% of primary bile acids that is not absorbed in the distal ileum can be reabsorbed or transported back to liver (where conjugation takes place) thanks to the bile salt hydrolases that are secreted by colon microbiota such as *Clostridium perfringens* and *Clostridium scindens*, which convert or deconjugate primary bile acids to secondary bile acids [182].

The study of relationships between commensal microbiota and host immunity is mostly assessed using GF animal models. Interesting results from early studies in these animal models have been reviewed by Zheng et al. [203]. These studies found that the absence of commensal microbiota is associated with bowel defects that are related to the lymphoid tissue structure and immune tasks [204]. The number of intra-epithelial lymphocytes bearing αβ T-cell receptors, which is reduced in GF mice compared to in colonized animals, immediately increases upon de novo colonization and almost reach the level found in conventional mice after 1 month [205]. Furthermore, the IgA antibodies play a pivotal role in humoral mucosal immunity protection, showing a substantial reduction in newborns and GF animals, which is rapidly restored by microbial colonization [206]. Th17 cells, which are abundant in the *lamina propria* in the small intestine, are potent immunomodulatory effector cells and are largely induced by segmented filamentous bacteria [207,208]. *Bacteroides fragilis* plays an important role in the maturation of immune system development in mice, including the correction of systemic T cell deficiencies and Th1/Th2 disparities in lymphoid tissues [209]. Moreover, gut microbiota alterations have been associated with an increased incidence of allergic and autoimmune disorders, and, in particular, its diversity during early-life colonization is critical to protect hosts from the induction of mucosal immunoglobulins E (IgE), an increase of which is linked to allergy susceptibility [210]. In short, host immune–microbiota interactions are a critical factor in early life and may even have a long-lasting impact on multiple immune arms that contribute to immune homeostasis and susceptibility to infectious and inflammatory diseases later in life.

The intestinal microbiota is also related to colorectal cancer (CRC), one of the most common cancers. Because microbiota alterations can be detected at the earliest stages of CRC, these alterations may be used as biomarkers for early CRC detection. Moreover, as previously mentioned for psychiatric disorders, modulating the intestinal microbiota, in this case by means of dietary strategies, may also be a new strategy for CRC prevention [211,212]. The consumption of probiotics and/or prebiotics may be another strategy to modulate the microbiota. Some chemical-induced animal model studies have reported that probiotics exerted a significant protective effect against CRC. *F. prausnitzii*, a potential probiotic, produces hydrophobic microbial anti-inflammatory molecules that can downregulate the NF-kB pathway in intestinal epithelial cells and can prevent colitis in animal models [213]. Treatment with a mixture of probiotics (*L. plantarum*, *L. acidophilus*, and *B. longum*) in CRC patients increased the amount of cell junction proteins, thereby improving intestinal mucosal barrier integrity [214]. One probiotic intervention study revealed that patients with CRC who received *B. lactis* Bl-04 and *L. acidophilus* NCFM had an increased abundance of butyrate-producing bacteria, such as *Faecalibacterium* and *Clostridiales spp*., and a decreased abundance of CRC-associated genera, including *Fusobacterium* and *Peptostreptococcus* [215]. Hence, several studies have reported the positive effects of probiotic use in CRC, including reduced a incidence of diarrhea, enhanced gut barrier integrity, and reduced inflammation [216,217,218].

Bacteriophages, which play an important role in the structuring of the human gut microbiome in terms of composition and function should not be overlooked. They constitute most viral particles in the aforementioned complex microbial ecosystem: a large-scale identification of viral genomes was recently performed from 11,810 publicly available human stool metagenomes, forming a metagenomic compendium of around 200,000 viral genomes as a result, of which more than 75% represented double-stranded DNA phages. These viral genomes represented considerable viral group diversity, which had been largely uncharacterized previously [219].

### 3.2. Endogenous Retrovirus in Human Genome: Between Disease and Evolutionary Symbiosis

To these fatal characters, the pathogenic bacteria, we must add other even smaller and no less fatal characters, viruses (flu, ebola, zika, hepatitis, HIV, herpes, smallpox, coronaviruses such as SARS-CoV, MERS, SARS-CoV-2, etc.), and it seems that little good can be said about them. In fact, the British biologist Peter Medawar and his wife Jean stated that “*No virus is known to be good: it has been well said that a virus is bad news wrapped in proteins*”; however, it is clear that they are wrong because the “villains” are only a few of the thousands if not millions that exist and that do not bother us. In fact, some of them even do us favors, just as the bacteria comprising our microbiota do. The diploid human genome comprises more than six Giga base pairs (bp) [220] and contains an extremely high level of genetic information, including thousands of genes [221]. Notably, around 8% of the human genome comprises DNA of viral origin [222], with almost 100,000 elements being from human endogenous retroviruses (HERV) and fragments, which have been incorporated throughout our evolution [223]. These elements represent an integral part of genome evolution and human pathology and physiology; they are pieces of our genetic puzzle that have been giving us advantages, allowing us to be who we are today, and they will surely continue to improve us. In fact, very conservative estimates indicate that almost 80% of the human genome is made up of repetitive elements that are produced by retro transcription [224]. By its nature, a virus is a parasite, but sometimes, this parasitism is more similar to a symbiosis, a relationship that benefits both the visitor and the host, in the same way that fire can be good or bad. In the case of viruses, it seems that they are less frequently bad than they are good, and it all depends on the virus and the situation. The endogenous retroviruses (ERVs) are remnants of germ line infection by exogenous retroviruses that occurred millions of years ago [223,225]. Retroviruses are made up of an RNA genome. Once it has entered the target cells, the viral RNA is retro-transcribed into a double-stranded DNA molecule. Thanks to a viral integrase and its interaction with cellular and viral proteins, this DNA is integrated into the cellular genome, forming what is known as a “provirus”. The viral proteins and genome will be expressed from this inserted proviral DNA, which supports the production of new viral particles [226]. In the case of endogenous retroviruses, the viral DNA was maintained over the course of evolution, sometimes becoming involved in it. Vertebrates share similar ERVs in terms of both sequence and positions, which is probably because of an exogenous retrovirus infection of a common ancestor [227].

The ERVs share the classical proviral structure with exogenous retroviruses, which is composed of two long terminal repeat (5′ and 3′ LTR) regions that contain the viral promoters and enhancers, the *gag* gene (coding for the structural capsid proteins), the *pro-pol* gene (that expresses the viral protease, integrase and retro-transcriptase), and the *env* gene (expressing for the envelope protein) [228]. ERVs have been subjected to numerous amplification and genome transposition events, giving rise to multiple copies of proviruses in the cellular genome [229], and some mosaic forms originating from retrovirus recombination have also been reported [230]. However, the action of the host editing systems and the genomic substitution rate have often made HERV proviruses defective, usually leaving a sequence that just expresses non-coding RNAs. Indeed, around 90% of genomic HERVs show deletions of the provirus due to homologous recombination between the flanking LTRs that leave a solitary LTR, an event that mainly seems to occur in recently integrated proviruses compared to in those from older times [231]. Nevertheless, in some cases, the provirus presents a residual protein coding capacity that was probably selected because it determines an evolutionary advantage that guarantees the preservation of the fittest host [228].

HERV-LTRs, which contain a promoter, enhancer, and polyadenylation signals, may influence the expression of the neighboring cellular genes. An LTR from the THE1D retro-element family has been reported as an alternative promoter for the expression of the β subunit of the IL-2 receptor in placental trophoblasts, where it is probably involved in decidual natural killer (NK) cell differentiation and proliferation [232]. HERV-K viruses probably colonized ancient primates as early as 55 million years ago. Of these, the HML-2 clade is the youngest and is the most highly preserved provirus that is capable of viral particle production [233]. Eighty-nine HML-2 elements have been observed in the complete genome, with thousands of “solo LTRs” being observed as well [234]. The higher integration of antisense solo LTR from the HML-2 in *RASGRF2* gene (known to be related to substance abuse due to its effect on dopaminergic pathway) has been observed in injection drug abusers [235]. HERV-K (HML-2) LTR is an enhancer of apolipoprotein C-I and it has been associated with the placental expression of the Endothelin B receptor [236]. The HERV-H polyadenylation signal has been linked to the expression of spliced transcripts from the Human Endogenous Retrovirus-H Long Terminal Repeat-Associating (HLLA) 2 and 3 human genes [237]. HLLA-2 is a B7 family member that is involved in the control of CD4 and CD8 T cell proliferation [238]. HERV-L LTR is the main promoter of β1,3-galactosyltransferase 5 in colon cells [239].

Notably, ERVs are frequently transcriptionally silent, mainly in germ line and early during embryogenesis [240], and although they could potentially express proteins, their maturation and biological activity may involve other cellular proteins and conditions, thus explaining their specific relationship to certain diseases [241].

A higher expression of HML-2 has been reported to be in the presence of HIV and cytomegalovirus infection [242,243]. The expression of HERV during HIV infection may promote the activation of the anti-HERV T cells that may promote the immunological control of HIV infection, as observed in elite controller patients [244].

As previously stated, HML-2 is the most preserved endogenous provirus. Two subtypes of proviral genomes have been reported: a complete sequence (type 2) and one sequence that is characterized by a deletion of more than 290 bp between *pol* and *env* (type 1). These different provirus subtypes encode two envelope-derived proteins respectively: the accessory proteins Rec (similar to HIV Rev protein), which is encoded by the type 2 proviruses, and Np9, which is encoded by the type 1 proviruses [245]. Both proteins are highly expressed in the presence of cancer and are considered to be potential oncoproteins. The Rec protein is reported to interact with a checkpoint protein by interfering with the androgen receptor activity in regulating the cell cycle in prostate cancer [246]. The Np9 protein may bind the MDM2 ubiquitin ligase, thus interfering with p53 activity [247], its upregulated expression in teratocarcinoma cells has been linked to increased cell migration, whereas its depletion has been linked to greater sensitivity to bleomycin and cisplatin [248]. HERV-K is highly expressed in the cortical and spinal neurons of patients with amyotrophic lateral sclerosis (ALS), where the *env*-encoded protein causes the neurites to bead and retract [249]. The disruption of HERV-K *env* using a CRISPR/Cas9 endonuclease from *Staphylococcus aureus* (saCas9) has been proposed as a therapeutic strategy for both cancer and neurodegenerative diseases such as ALS [250].

Although HERV are usually silent, some of them play a key role in mammalian evolution. Human trophoblast syncytialization is a process of cell–cell fusion that allows the formation of invasive *syncytiotrophoblasts*, which are multinucleated syncytia that form the outer layer of *placenta villi*, which are essential to ensuring the adequate transfer of compounds between maternal and fetal blood [251,252]. With the exception of egg-laying mammals (echidna and platypus), all mammals form a placenta. Human placenta (apes, monkeys, and rodents) is characterized by the penetration of trophoblasts into capillaries, ensuring this close and direct connection with the maternal blood [251]. This membrane also helps prevent the maternal immune system from attacking the fetus as if it were a foreign body [253]. Syncytin-1 is produced from the HERV-W *env*, which entered the primate genome approximately 25–30 M years ago, whereas Syncytin-2 is encoded from HERV-FRD *env*, whose integration in primates probably occurred more than 45 M years ago [254,255]. Both proteins bind two different transporters expressly into the membrane of the fusing partner cells (the Na-dependent neutral amino acid transporter 2 -ASCT2- and the major facilitator superfamily domain-containing protein 2 -MFSD2- for syncytin-1 and 2, respectively), thus causing some structural reorganization of the syncytin proteins that enable membrane fusion between the two cells [251]. Placenta formation is essential to guarantee the optimal fetus development during pregnancy, and the insertion of these ancient retroviruses may have provided the host with an evolutionary advantage that allowed the emergence of placental mammalian ancestors from egg-laying animals [256]. In this way, a new living being can undergo a maturation process within the mother’s body, guaranteeing the thermal stability of the developing living being while also allowing the mother to move, seek refuge from inclement weather, protect herself from predators, and search for food without abandoning her offspring—situations that are much more dangerous when maturation occurs in a nest, which is much more exposed to dangers from predators and even environmental changes; this very real advantage is thanks to a virus. Similar syncytin proteins that were derived from other HERVs have been reported in other placental mammals [251], providing a great example of how HERVs contribute to the evolution of mammals. In addition, HERV-K may also be involved in placentogenesis and pregnancy [257] since the expression of its transmembrane (TM) envelope protein was detected in villous and extravillous cytotrophoblast cells. The expression of this protein may contribute to the immune protection of the fetus due to the immunosuppressive properties of retroviral transmembrane envelope proteins. In fact, the TM of a HERV-K (HML-2) sequence was reported to inhibit T cell activation in a similar way to that of the HIV TM immunosuppressive domain, influencing cytokine release and immune gene expression [258].

Also relevant to mammal evolution is the protein Tp63, a homolog of the tumor suppressor p53 protein, whose expression is controlled by a HERV9 LTR, and it is expressed in spermatogenic precursors after DNA damage, where it suppresses cell proliferation and induces cell apoptosis [259,260]. The HERV9 LTR insertion happened during the separation of *Hominidae* from *Hylobatidae* (~15 M years ago)*,* and likely served to enhance the genomic stability of the male germ line, providing some much needed regulation and a prolonged fertility time frame [261]. Indeed, it acts as a specific germ-line promoter of this gene [261], and its pharmacological activation by histone deacetylase has been proposed as a treatment strategy for testicular cancer [262].

Another example of human genomic material that is derived from viruses is the *ARC* gene, which is expressed in response to neuronal activity in mammals and flies, although in each case, *ARC* seems to have arisen from different phylogenetic origins [263]. This gene is very similar to the retroviral *gag* genes that encode capsid proteins. In fact, the neuronal protein ARC, which acts as a regulator of synaptic plasticity [264], is evolutionarily related to the Gag proteins that are encoded by the Ty3/gypsy retrotransposon family [265], and they form virus-like capsid structures [266]. It has been suggested that this gene may play a key role in information storage within neural networks [264]. Another word for that is “memory”. In fact, ARC proteins pack information that is derived from experience in the form of RNA molecules, which is similar to viruses, as a kind of capsid that is made up of ARC proteins carries these messenger RNA molecules from a donor neuron to a receptor [263,267]. As stated above, it was suggested that the human *ARC* gene originated from a mobile genetic element of the Ty3/gypsy retrotransposon family, one of four families of retroelements and that is characterized by being flanked by LTR sequences [266], which indicates its retroviral origin. As reviewed by Parrish and Tomonaga [268], there is a growing group of metazoan genes of that are viral origin that harbor critical functions in the physiology of their “host” organisms, among which the *ARC* gene may be included.

Finally, HERVs have been proposed to play a role during exogenous viral infections, directly interacting with them, as reviewed by Grandi et al. [228]. This interaction may be beneficial to viruses if they cause the upregulation of HERV expression. In this case, immune triggering due to HERV products may be increased, possibly causing the complementation of defective viruses and recombination events, especially in retroviral infections [269]. However, HERVs also show protective effects against exogenous infections, mainly against retroviruses that share the same identity in their protein and nucleic acids through the restriction of different parts of the viral cycle. In the review by Grandi et al. [228], three major protective effects of HERV are presented, and HIV is used as an example: the interference and blocking of HIV cellular receptors by the binding of the HERV proteins or pseudoparticles to the same receptor; the formation of dsRNA through the interaction resulting from the complementarity of the HERV mRNAs with HIV RNA, which could be detected as a PAMP (pathogen-associated molecular pattern) by cellular innate immunity sensors; and the identity between HERVs and HIV proteins, which may lead to complementation events, possibly affecting the assembly and release of viral particles. Another interesting example of the protective effects of HERV against viral infections that was commented upon by Grandi et al. [228] is the upregulation of HERV-K (HML-2) Rec proteins during embryogenesis. This upregulation leads to the specific stimulation of the interferon-induced viral restriction factor IFITM1 in epiblast and embryonic stem cells [270,271], suggesting that they may elicit an innate anti-viral response [270].

### 3.3. Microorganisms Helping Us Even before We Were Aware of Their Existence. Biotechnological Allies for 10,000 Years

As described in the previous sections, in which we present the positive contributions of microorganisms, they are the progenitors of all life on Earth and therefore of our species. In the section dedicated to the microbiota, we showed that the microbiome harbors 50-fold to 100-fold more genes than the host [182], and these genes control essential functions that are required to keep us alive [181]. However, there is another aspect in the beneficial collaboration of microorganisms that we do not usually take into account; long before humans were aware of their existence, microorganisms were already being exploited to satisfy the needs and desires of humans, such as by preserving milk, fruits, and vegetables and by improving our quality of life with drinks, cheeses, bread, and pickled foods and vinegars. The utilization of microorganisms as biotechnological tools to achieve those goals was reviewed by Demain et al. [180]. According to this work, fermentation by means of yeasts was already used in ancient times; for example, in Sumer and Babylon, it was used to make beer earlier than 7000 BC, and in ancient Egypt, it was used to leaven bread in around 4000 BC. Indeed, bread was a particularly important food in ancient Rome, which had more than 250 bakeries, meaning that humans were making leavened bread by 100 BC. Moreover, wine was made in China as early as 7000 BC [272] and in Assyria in 3500 BC [273], and references to it can be found in ancient texts such as the *Book of Genesis*. Ancient people also made use of bacteria to obtain vinegar, for example, through the fermentation of alcohol from fruit or plant juices that had previously been fermented by yeast [274]. According to the work by Demain et al. [180], even the Talmud cites vinegar, which the Assyrians used as a treatment for chronic diseases of the middle ear, and Hippocrates used vinegar for medicinal purposes in 400 BC. For thousands of years, human beings have made cheese with different species of fungi (molds) and bacteria, giving rise to different characteristic flavors [275]. In fact, moldy cheese, meat, and bread have been used in folk medicine to heal wounds and, in general, the curative properties of molds have been widely used since antiquity [276]. As a preservation method for milk, which spoils easily after being obtained from milk-producing animals, it was discovered many centuries ago that it could be fermented and acidified by lactic acid bacteria to make yogurt as well as into kefir and kumiss using the *Kluyveromyces* species [180,277,278]. Microorganisms can also be harmful for the production and preservation of food. For example, in the 19th century, the Lille distillers in France asked Pasteur to find out why the contents of their fermentation vats were turning sour. Indeed, the distillery managers were facing economic problems due to reduced alcohol yields, increases in sour wine, some of which even turned to vinegar and, in addition, vinegar failing to form when it was needed, as lactic acid was being produced instead. Pasteur observed that, in addition to yeast cells in the fermentation mixture, bacteria were present when lactic acid was produced. These bacteria seemed to be responsible for turning the fermentations sour since the complex organic compounds that were detected could not be explained by the simple catalytic breakdown of sugar and must have been produced by living cells. To prevent this bitterness, Pasteur applied a mild heat treatment, which later became known as pasteurization [180,279]. The millenary use of fermentation, which we can consider as a kind of domestication of these invisible beings, even before we were aware of their existence, has allowed for the development of what we now call enzymology, and in practice, it can be considered to be the origin of biochemistry. More recently, human beings began to use microorganisms as biotechnological tools for other purposes in addition to food and medical ones. During World War I, the need for glycerol, which is used to make ammunition, resulted in the beginning of its industrial production through the application of the yeast *Saccharomyces cerevisiae,* which was able to convert sugars into glycerol [180,280].

Over the few past decades, these initial empirical applications of fermentation have resulted in a genuine scientific revolution: biotechnology, which has been enormously beneficial for humanity. The word “biotechnology” was coined around 1919 by the Hungarian agricultural engineer Károly Ereky, who used the term in the title of his book *Biotechnologie der Fleisch-, Fett-, und Milcherzeugung im landwirtschaflichen Großbetriebe* (Biotechnology of meat, fat, and milk production in large-scale agricultural industries), which was based on the 19th century formulations of the theory of work and management, philosophy, economics, biology, and chemistry. Ereky envisaged the use of biology to convert raw materials into useful products, resulting in a new industrial revolution. His vision, which would soon be applied to microorganisms more than it would be macroorganisms, became popular with agrobiologists, chemists, and engineers [180,281,282]. As a well-known example, we also have Fleming’s discovery, which led to penicillin, the first successful chemotherapeutic agent to be produced by a microbe. In 1928, Fleming noticed an invading fungus on an agar plate that had a zone around it where the bacteria seemed to be unable to grow. He used that “raw material” to identify that mold as belonging to the *Penicillium* genus and obtained an extract from it whose active agent was penicillin, which he considered to be a “useful product”. However, penicillin was an unstable compound, and Fleming was not able to purify it from the extract. Thus, it was not until more than a decade later and under the eminent pressure of World War II that it was given its first clinical use, which required remarkable optimizations of *Penicillium notatum* (Fleming’s strain), which produced only traces of penicillin: changing the *Penicillium* strain to *Penicillium chrysogenum* and modifying the culture medium made it possible to increase the yield of penicillin a hundred-fold within two years and meant that it could be used to treat those who had been wounded in battle by 1943 [283,284]. Subsequent improvements in penicillin production included genetic manipulations [283], which allowed the product of this microorganism to be used to save millions of lives both on and off the battlefield. The discovery of penicillin led to the development of many other semisynthetic β-lactam antibiotics, many of which were derived from the 6-aminopenicillanic acid, which has been widely prescribed since 1941, making it one of the first clinical uses of penicillin [285]. In fact, the discovery and the development of β-lactam antibiotics has been considered as “*one of the most powerful and successful achievements of modern science and technology*” [283]. In addition, penicillin paved the way for the development of many other antibiotics, and currently, more than 100 antibiotics are used against infections in humans, animals, and plants [180]. Not all of those antibiotics are produced by molds. In the 1940s, Waksman and his students discovered many new antibiotics that could be produced by actinomycetes, a group of filamentous soil bacteria, which led to the discovery of streptomycin in 1944, the first effective cure for tuberculosis [286,287]. For some reason, actinomycetes are surprisingly prolific in the amount of antibiotics that they can produce. In fact, they produce most of the antibiotics that are currently used today. In particular, according to data published in 2016, 80% of antibiotics are sourced from a single genus, *Streptomyces,* and other rare actinomycetes, and only 20% are produced by fungi [288]. The world production of antibiotics is estimated at more than 60,000 tons per year [289,290]. This growing demand for antibiotics has spurred the improvement of antibiotic producing strains, as genetic recombination, which was practically ignored at the end of the 1970s, was improved in 1985 and became the engine for the development of multiple technologies that are now very familiar to us, changing the situation considerably: (i) rearrangement mutagenesis, (ii) genetically engineered deletions and duplications, and (iii) genetic recombination by protoplast fusion and plasmid transformation [180]. It was revealed that the genes that encode most of the antibiotic biosynthetic pathways were grouped into operons as described for the *Streptomyces coelicolor* model system [291], thus facilitating the transfer of entire pathways from one organism to another. Together, this gave rise to the birth of “combinatorial biosynthesis” in 1985, which used genetic engineering to modify antibiotic biosynthetic pathways and became a widely used technique for the discovery of new hybrid drugs [180,292], resulting in the consolidation of g recombinant DNA (rDNA) technology and the discovery of new antibiotics [293,294,295]. In addition to combinatorial biosynthesis, large-scale antibiotic production has also promoted the extensive use of other new genetic techniques. According to Demain et al. [180], progress in strain development has also involved metabolic engineering, the use of rDNA for the directed improvement of cellular properties through the modification or introduction of biochemical reactions in metabolic pathways [296]; directed evolution that consists of promoting protein evolution in the laboratory by generating genetic variability and then identifying those with the desired properties [297]; and molecular breeding, which consists of genetic manipulation to improve traits of interest, similar to the artificial selection of agriculturally important animal and plant populations [298]. In addition to antibiotics, rDNA led to the development of many more recombinant products. The first recombinant biotechnology products, such as interferon (IFN), which is produced by rDNA [299], were the direct result of the basic research that led to these new technologies. In addition, taking advantage of the ability of bacteria to produce hormones, Genetech, the first genetic engineering company, marketed human insulin in 1982, making it the first commercially manufactured product to use rDNA [300]. By making use of these technologies, the same company marketed or licensed other clinically relevant recombinant products such as human growth hormone, the hepatitis B vaccine, the blood clotting factor VIII, etc. Thus, in the era of rDNA, biotechnology makes it possible to produce molecules such as hormones, IFNs, interleukins, antibodies, etc., for the medical treatment of many diseases, a tremendous advance that had was not possible before.

Scientific breakthroughs and technological progress allowed development of new methods and tools that enhanced the expansion of recombinant technologies. Some of the most important rDNA methods as reviewed by Demain et al. [180] and include the analysis of the sequence and structure of DNA, RNA, and proteins, the synthesis of short DNA molecules, and the identification and purification of DNA molecules that encode pharmaceutically active proteins and that are coupled to the introduction of this DNA (also of human origin) in bacteria and protein expression. There are countless beneficial applications of this technology that have emerged from these invisible beings. Additionally, we must not forget the molecular biology techniques that have been developed based on the use of multiple microorganism components (such as *Eschericcia coli*): polymerases, restriction enzymes etc., or that the most relevant technique in molecular biology, the polymerase chain reaction (PCR) described by K B Mullis [301], which uses polymerases that have been obtained from hot spring bacteria, mainly of the genera *Thermus*, *Thermococcus,* and *Pyrococcus* (*Thermus aquaticus*, *Thermus thermophilus*, *Pyrococcus furiosus*, etc.) [302]. It is impossible to imagine our current world without all of these biotechnological processes, even when we did not even know that they were biotechnological processes, such as fermentation, which we have used for almost 10,000 years. Once again, microorganisms reveal themselves to be vital allies of the human race.

## 4. Can Microorganisms Structure Population Genetics?

With the data on microbiota and endogenous viruses in hand, we should give microorganisms a chance, should not always label them as evil beings, and should even consider their role as biotechnological allies. We have seen that “as villains”, a reputation that they gained by means of terrible pandemics, these invisible beings are able to transform their environment and therefore our history. However, we might wonder whether these pandemics may even structure the genome of the human populations that they affect. This is a speculative question that still has no direct answer, but there is some very recent evidence that this may be the case. One of these pieces of evidence is the report by Laayouni et al. [27]. This study indicates that in recent historical periods in Europe, serious epidemic episodes such as the Plague, smallpox, or influenza have taken place, and puts forward the hypothesis that these infections have shaped the immune system of modern populations, probably enriching it in terms of gene polymorphisms that have a protective role against these deadly infections. As proof of this interesting hypothesis, the study identifies signs of convergent evolution of the immune system in two populations in Romania with different genetic ancestry: autochthonous Europeans and the Rroma, the latter of which come from emigrations from northern India and who have lived in the same geographical area and have been exposed to similar infections over the past millennium. For example, this study identifies strong signals of adaptive selection in the Toll-like receptor 1 (*TLR1*)/*TLR6*/*TLR10* gene cluster in autochthonous Europeans and in the Rroma. At the same time, single-nucleotide polymorphisms (SNPs) in this gene cluster were shown to modulate cytokine responses that were induced by *Yersinia pestis* (the causal agent of the Plague), so it is likely that this infection played an important role in its selection. On the other hand, these genes do not show this selection in a northwestern Indian population, the ethnic origin of the Rroma people, where these great Plague pandemics did not take place.

A similar phenomenon has been proposed as an explanation for the different ethnic and geographic distribution of the CCR5-Delta32 receptor variants (deletion) that inactivate the HIV-1 coreceptor in lymphoid cells, resulting in strong resistance to HIV-1 infection. This variant is found in Caucasian populations in Eurasia at frequencies of 0–14%, while it is absent in Native Africans, Native Americans, and East Asians. In addition, a strong linkage disequilibrium was observed between the *CCR5* locus and two microsatellite loci in Caucasian populations, suggesting an estimated age for the allele of 700 years (between 275 and 1875), which may indicate its positive selection during epidemics in Europe (such as Plague or smallpox pandemics that have taken place over the past 1000 years), consequently reducing the susceptibility of modern Europeans to HIV-1 infection compared to Africans [303]. However, despite the absence of the CCR5-Delta32 allele outside of Europe and the long-range linkage disequilibrium at the locus, by re-evaluating this evidence in another study [304], performing high-density SNP genotyping around the *CCR5* locus in multiple populations and analyzing the data with large genomic comparison datasets and revised physical and genetic maps, it has been found that the pattern of genetic variation in CCR5-Delta32 is consistent with neutral evolution, as it does not clearly stand out as exceptional relative to other alleles at the same locus or in other loci throughout the genome. Furthermore, the CCR5-Delta32 allele has been estimated to have arisen more than 5000 years ago [304,305], long before the epidemics in Europe that were supposed to have positively selected it.

In relation to the tremendous SARS-CoV-2 pandemic that we are currently suffering, something similar can be speculated. Recently, Ellinghaus et al. [306] reported the association of certain genes with the severity of the disease caused by that virus (COVID-19), which shows a considerably variable clinical outcome among patients. In this study, cross associations were detected with the SNPs rs11385942 at the 3p21.31 locus and with rs657152 at the 9q34.2 locus. At the 3p21.31 locus, the association signal spanned the genes *SLC6A20*, *LZTFL1*, *CCR9*, *FYCO1*, *CXCR6,* and *XCR1*, constituting a locus of genetic susceptibility in patients with COVID-19 with respiratory failure. The association signal at the 9q34.2 locus coincided with the ABO blood group locus, showing a higher risk in blood group A and a protective effect in blood group O. This last observation seems to agree with the classical report by Pettenkofer et al. [307], hypothesizing a relationship between the distribution of the ABO blood group genes and the history of major pandemics. Pettenkofer already reported the nonA groups as protectors, which was perhaps due to the presence of the anti-A antibody, which is able to block infectious agents. Similar results were reported in Iceland [308], suggesting that the low frequency of group A and the high frequency of group O in the population of that island could be due to the disadvantage of being a carrier of group A during a severe smallpox epidemic. It was also observed to be associated with the smallpox epidemic in India from 1965–66 [309]. Therefore, it seems that the condition of the microorganisms affects the distribution of blood groups; that is, the genetic load of the communities where they act.

Shortly after the Ellinghaus study was published, Zeberg et al. [28] reported that the genes on locus 3p21.31 in chromosome 3 that were linked to genetic susceptibility for respiratory failure in patients with COVID-19, which were detected in the study by Ellinghaus, are related to a group of genes inherited from Neanderthals. This human species evolved separately from the ancestors of modern humans in Africa in western Eurasia about half a million years ago [310]. The Neanderthals adapted to the Eurasian environment as well as infectious diseases, strong selective factors that may (at least partly) have differed between sub-Saharan Africa and Eurasia. Neanderthals, as well as Denisovans, their Asian sister group, became extinct about 40,000 years ago [311]. Therefore, both human species coexisted in Eurasia with our ancestors, resulting in some interbreeding with them. In this regard, the genome of people from outside of Africa carries 1.5–2.1% of DNA from Neanderthals [310], including several genetic variants that affect genes that are involved in immunity [312,313,314], such as the Toll-like receptor gene variants that decrease the susceptibility to *Helicobacter pylori* infections and the risk for allergies [315]. Similar to this, some proteins that interact with RNA viruses have been shown to be encoded by Neanderthal genes [316].

According to Zeberg’s report, when infected by SARS-CoV-2, each copy of this Neanderthal haplotype approximately doubles the risk of requiring intensive care. This haplotype reaches carrier frequencies of up to approximately 65% in South Asia and approximately 16% in Europe, whereas it is almost completely absent in East Asia [28]. In this regard, as suggested by Enard and Petrov [317], different types of viruses may have exerted different selective pressures during human evolution. The different carrier frequencies of that Neanderthal haplotype in different human populations may be linked to the presence of coronavirus pandemics in ancient times in East Asia [318], where, as it has been shown in recent years, these infections have been emerging as zoonosis (SARS-CoV, MERS). We may therefore speculate that these antique coronavirus infections would have affected the carriers of these Neanderthal-related risk genes much more severely than the non-carriers, resulting in a dramatic decrease in their proportion in the affected population and explaining their current practical absence in East Asia in contrast to their high proportion in South Asia, where coronaviruses would not be as frequent. In fact, individuals of Bangladeshi origin (South Asia) in the UK have a two-fold higher risk of dying from COVID-19 than the general population [319]. Browning et al. [320] reported that anatomically modern humans interbred with Denisovans, suggesting that Neanderthal haplotypes have been positively selected in Bangladesh, perhaps as protection against other pathogens. Regarding this last suggestion, using data from a genome-wide association study of the Genetics of Mortality in Critical Care consortium on host genetic variants associated with critical illness [321], Zeberg and Pääbo [29] recently reported another Neanderthal inherited haplotype that, in contrast to the previously described haplotype [28,306], is protective against severe disease, but that it has a more moderate effect (∼22% reduction in relative risk of needing intensive care, thus becoming severely ill with COVID-19 when infected by SARS-CoV-2). Among the seven loci that were identified in the genome-wide association study [321], that haplotype is located on locus 12q24.13, which is linked to the SNP rs10735079. According to data from the 1000 Genomes Project [322], this haplotype occurs at substantial frequencies, 25–30% in most populations in Eurasia, while it is almost absent in African populations that are located south of the Sahara. Among ancient human genomes in western Eurasia, the frequency of the protective Neanderthal haplotype may have increased between 20,000 and 10,000 years ago and again over the past 1000 years [29]. This haplotype contains parts or all of the *OAS1*, *OAS2*, and *OAS3* IFN-stimulated genes, which encode oligoadenylate synthetases [29]. These enzymes are activated by double-stranded RNA and regulate the early phase of viral infections by leading to the synthesis of 2′-5′ oligoA and RNase L activation. The RNAseL cleaves dsRNA, which is recognized by the immune system pattern recognition receptors, activating other antiviral pathways [323].

FMF, a common inherited autoinflammatory disease, is another example on how serious epidemic episodes (the Plague in this case) may have contributed to the structuring the genome of human populations, something that was commented upon in Section 2.2.2. How did the Black Death change history? This disease is caused by recessive mutations in the gene that encodes the inflammatory response protein known as pyrin. This disease mainly affects populations in the eastern Mediterranean basin, largely affecting Jews, Turks, Armenians, and Arabs [62]. After a bacterial infection, pyrin forms a complex with the inflammasome, causing the release of pro-inflammatory cytokines and inducing pyroptosis as a defense mechanism against infection [324]. To counteract these host responses, pathogenic *Yersinia* species secrete YopM, a virulence factor that inhibits the activation of the inflammasome by pyrin [325]. Park et al. [30] have recently reported that FMF-causing mutations are associated with an increased release of pro-inflammatory cytokines from human leukocytes and the increased survival of mice after *Y. pestis* infection. The bacterial factor YopM appears to activate host kinases that negatively regulate the activation of the pyrin inflammasome through phosphorylation [325]. Because the pyrin variants that cause FMF lead to the excessive activation of the inflammasome, due to gain-of-function mutations in the *MEFV* gene [326], this excessive activation compensates for the inhibitory role of YopM that enables cellular pyroptosis, resulting in resistance to infection [30]. FMF mutations are relatively common in middle eastern regions [62], where the plague was endemic [327], which suggests that these mutations have been selected to counteract the virulence strategy of *Y. pestis*. Parks’ study [30] suggests that FMF variants have increased in frequency rapidly in the last few thousand years, probably emerging 1800–2600 years ago. This is consistent with the action of recent positive selection, such as the first major Plague pandemic documented 1500 years ago (the Justinian Plague, 6th century) [8,37,38], conferring a certain selective advantage on the people of the Middle East by increasing their resistance to the Plague. The selective pressure to generate the observed mutation carrier frequency requires geographic convergence between the mutations and a selector agent, such as an infection responsible for substantial population mortality; a well-known example is the case of the hemoglobin S, which is responsible of Sickle Cell Anemia and protection against death from malaria in Sub-Saharan African Populations [328]. Continuous Plague pandemics since the Justinian Plague (such as the terrible Black Death during the 14th century) have probably improved the selection for FMF mutations in the European population. In fact, the extremely sad pogrom episodes against Jews, in which these groups were massacred after being frequently accused of being responsible for the Plague, may be related to lower mortality in these communities due to the high prevalence of FMF mutations in these populations [62].

## 5. Final Remarks

Throughout this review, we have discussed several examples of how microorganisms shaped human beings as well as their enormous impact on the history of civilizations. We presented them as “villains” when this is translated into something harmful from a human point of view, such as the great pandemics throughout universal history, or as “friends” when their multiplication results in beneficial effects from the human perspective, such as all of the benefits that are provided by our gut microbiome as well as those bestowed upon us by endogenous retroviruses, or microorganisms which have contributed to multiple biotechnological processes. Nevertheless, the reality is that the sole purpose of bacteria and viruses is to multiply in a certain environment, and both pathogens and symbionts use the same mechanisms and conduct a similar molecular dialog with their hosts to achieve that goal.

Great examples of this are the bacteria of the genera *Agrobacterium* and *Rhizobium*. Both of them attach to the surface of plant cells and transfer genetic material into them: *Agrobacterium* transfers part of its tumor-inducing plasmid to the host plant cell through its transferred-DNA, causing plant diseases, including crown gall, cane gall, and hairy root [329]. *Rhizobium* most often enters through root hairs and induces nodule formation in the plant cortex, where those bacteria eventually enter the cytoplasm of the nodule cells and differentiate into bacteroids. However, instead of causing tumors, they form a structure called a symbiosome, together with bacterial and plant-derived membranes, where plants provide carbon and energy, and the bacteroids fix N_2_, making it available to plants in the form of ammonia, which can be used to synthesize amino acids [330]. *Rhizobium* is a friend, and *Agrobacterium* is harmful, but they both use similar mechanisms and conduct a similar molecular dialog with their hosts. In Section 3.2 of this review, Endogenous Retrovirus in Human Genome: Between Disease and Evolutionary Symbiosis, we also discussed the major protective effects of HERVs against exogenous viral infections by producing analogous products to those of these exogenous infections, such as the proteins or pseudoparticles that bind to the same receptor or that are complementary to the exogenous ones (which are able to interfere with the assembly and release of viral particles) [228].

Thus, we may conclude that classifying microorganisms as “villains” or “friends” reflects the fact that we are self-centered beings and that we usually look at nature with the wrong pair of glasses. Microorganisms teach us to be humble, especially through the current COVID-19 pandemic.

## Figures and Tables

**Table 1:** Epidemics brought to America by European colonizers from 15th to 18th century [10,43].

Year	Region	Infection	Observations/Consequences
1495	Antilles	Measles (viral infection, Paramyxoviridae)	
1508	Central America	Measles (viral infection, Paramyxoviridae)	
1500	Antilles/Central America	Yellow fever (viral infection, Flaviviridae)	Possible infection by mosquitoes brought from Cape Verde
1518	Mexico/Central America/Peru/Santo Domingo	Smallpox (viral infection, Poxviridae)	In Santo Domingo of a million inhabitants in 1518, only 500 remained in 1548
1519–1520	Peru	Smallpox (viral infection, Poxviridae)	200,000 deaths before the arrival of Pizarro
1532	Honduras and Nicaragua	Rubella (viral infection, Matonaviridae)	Almost half the population died
1558	River Plate	Smallpox (viral infection, Poxviridae)	
1563	Brazil	Smallpox (viral infection, Poxviridae)	
1585	Caribbean and Florida	Typhus (bacterial infection, Rickettsiaceae)	Francis Drake attack, ship’s crew infected in Cape Verde
1616–1619	New England	Typhus (bacterial infection, Rickettsiaceae)	90% mortality
1630	Canada (great lakes)	Smallpox (viral infection, Poxviridae)	Kills half of Iroquois Indians
1635	Canada	Rubella (viral infection, Matonaviridae)	
1669	Brazil (Rio de Janeiro)	Smallpox (viral infection, Poxviridae)	
1738	Canada (great lakes)	Smallpox (viral infection, Poxviridae)	Cherokee Indians almost annihilated
1760	Canada (great lakes)	Smallpox (viral infection, Poxviridae)	Catawba and Omahas Indians almost annihilated

## Data Availability

Not applicable.
